# Integrated GC–MS Phytochemical Profiling and Bioactivity Assessment of Leaf and Fruit Extracts of *Psidium cattleyanum* (Cherry Guava)

**DOI:** 10.3390/molecules31172984

**Published:** 2026-08-26

**Authors:** Shashini S. Werellagama, Ranjith K. B. Edirisinghe

**Affiliations:** Department of Chemical Sciences, Faculty of Applied Sciences, Rajarata University of Sri Lanka, Mihintale 50300, Sri Lanka; ranjith_e@rjt.ac.lk

**Keywords:** GC–MS, *Psidium cattleyanum*, phytochemical screening, TPC, TFC, DPPH IC_50_, brine shrimp lethality assay, continuous solvent extraction

## Abstract

*Psidium cattleyanum* (Cherry Guava, family Myrtaceae) is traditionally used for its medicinal and nutritional value; however, comparative chemical and bioactivity profiling of its leaf and fruit extracts remain limited. In this study, a sequential solvent extraction using hexane, dichloromethane, and methanol was employed to characterize the phytochemical composition. The combined extracts were analyzed by gas chromatography–mass spectrometry (GC–MS). Phytochemical screening, total phenolic content (TPC), total flavonoid content (TFC), DPPH radical scavenging assay, and brine shrimp lethality bioassay were conducted. GC–MS analysis identified terpenoids enriched in leaf extracts, while fruit extracts exhibited relatively higher contribution of lipid-derived compounds. Phytochemical screening revealed a higher diversity of secondary metabolites in leaf extracts compared to fruits. The leaf extract exhibited significantly higher TPC (31.96 ± 0.05 mg GAE/g) and TFC (51.43 ± 0.22 mg CE/g) than the fruit extract (8.87 ± 0.11 mg GAE/g and 1.98 ± 0.07 mg CE/g, respectively), correlating with stronger antioxidant activity (IC_50_ = 47.10 ± 0.06 ppm for leaves, 357.30 ± 0.64 ppm for fruits). Cytotoxicity assessment revealed mild to moderate bioactivity, with LC_50_ values of 522.14 ± 17.00 µg/mL (leaf) and 402.60 ± 9.00 µg/mL (fruit). Overall, this study highlights *Psidium cattleyanum* extracts as sources of antioxidant-associated phytochemicals exhibiting preliminary biological activity and provides a basis for future phytochemical and bioactivity investigations.

## 1. Introduction

*Psidium cattleyanum*, commonly known as Cherry Guava or Strawberry Guava, belongs to the Myrtaceae family and is a medicinal plant used in traditional healing practices. This evergreen shrub or small tree is native to South America, particularly Brazil, and has been introduced to various tropical and subtropical regions across the globe [1]. The plant is characterized by its dark green, oval-shaped leaves and produces small, round fruits with a sweet, strawberry-like flavor. Although valued for its edible fruits and ornamental qualities, *P. cattleyanum* has become invasive in some introduced areas owing to its rapid growth and ability to outcompete native vegetation [2]. Mass spectrometry has been employed to analyze the chemical composition of fruits of *P. cattleyanum*. Previous studies have shown that *Psidium cattleyanum* fruits contain diverse bioactive compounds, including carotenoids and anthocyanins such as β-carotene, β-cryptoxanthin, malvidin-3-glycoside, and cyanidin-3-glycoside, which are associated with antioxidant activity [3].

Chemical characterization of plant extracts is a crucial step in understanding their potential therapeutic properties and biological activities. Gas chromatography–mass spectrometry (GC–MS) is a powerful analytical technique that provides valuable insights into the composition of extracts from leaves and fruits. This method allows for the identification and quantification of individual compounds, enabling researchers to compare the chemical profiles of different plant parts and detect potential bioactive substances. GC–MS is a commonly employed analytical method for identifying volatile and semi-volatile metabolites in plant extracts, offering chemical profiles that are valuable for comparative phytochemical studies. Nonetheless, many highly polar substances, such as flavonoids, glycosylated phenolics, and tannins, typically require derivatization before they can be analyzed directly by GC–MS. As a result, GC–MS only partially reflects the phytochemical makeup of plant extracts and should be considered in conjunction with other phytochemical and bioactivity evaluations.

Plants of the family Myrtaceae are widely recognized as rich sources of bioactive secondary metabolites, particularly phenolic compounds, flavonoids, terpenoids, and essential oils, which contribute to their antioxidant, antimicrobial, and pharmacological properties [4]. Within the genus Psidium, several studies have reported high phenolic content and strong antioxidant activity in different plant parts, especially leaves and fruits [5]. Previous investigations on *Psidium cattleyanum* and related species have mainly focused on targeted compound classes, single-solvent extractions, or isolated bioactivities, often without integrating comparative volatile and semi-volatile phytochemical profiling with functional bioassays [3]. Moreover, comparative studies simultaneously evaluating leaf and fruit tissues using pooled multi-solvent extraction strategies combined with GC–MS analysis and bioactivity assessment remain limited. This highlights the need for an integrated approach to better understand organ-specific phytochemical composition and associated biological potential.

Sequential solvent extraction using solvents of increasing polarity has been commonly employed to obtain broad-spectrum phytochemical profiles prior to GC–MS analysis [6]. The present study contributes additional comparative phytochemical and bioactivity data for *Psidium cattleyanum* leaf and fruit extracts using a unified analytical framework. Previous studies have often relied on single-solvent extraction strategies or focused on isolated bioactivities without correlating phytochemical composition with functional outcomes [1]. Although individual phytochemical and bioactivity studies have been reported for *Psidium* species, comparative datasets integrating these approaches for both leaf and fruit extracts of *P. cattleyanum* remain limited. The aim of this study was to comparatively evaluate the phytochemical composition and bioactive potential of *Psidium cattleyanum* leaf and fruit extracts using a pooled sequential solvent extraction approach to broaden metabolite coverage and obtain a chemical fingerprint of *P. cattleyanum* leaf and fruit extracts by combining GC–MS-based qualitative profiling with phytochemical screening, total phenolic and flavonoid quantification (TPC & TFC), DPPH radical scavenging activity, and brine shrimp lethality bioassay. The present work provides a comparative evaluation of phytochemical characteristics and bioactivity responses of leaf and fruit extracts of *P. cattleyanum*.

Although GC–MS-based phytochemical profiling of Psidium species has been previously reported, most studies have focused on single plant parts, single-solvent extracts, or isolated bioactivity assays without integrating comparative metabolite distribution with functional bioassays. Furthermore, limited attention has been given to organ-specific metabolic specialization between leaves and fruits under a unified extraction and analytical framework. Comparative metabolomic approaches are valuable for understanding how different plant tissues allocate secondary metabolites and how these variations relate to biological activity. Therefore, a combined chemical–biological profiling approach is required to move beyond descriptive phytochemical cataloguing toward functional interpretation of metabolite variation.

## 2. Results and Discussion

### 2.1. Phytochemical Screening

Phytochemical screening revealed marked differences between leaf and fruit extracts of *Psidium cattleyanum*. The leaf extract exhibited a broader diversity of secondary metabolites, notably alkaloids, tannins, phenols, flavonoids, and terpenoids, whereas the fruit extract primarily contained flavonoids, phenols, steroids, and terpenoids [Table S1, Supplementary Materials]. Representative compounds corresponding to these groups, later confirmed by GC–MS analysis, include phenolic constituents such as terpenoids such as β-caryophyllene, phytol, and sterols such as γ-sitosterol. These findings indicate organ-specific differences in metabolite accumulation, which may influence the observed bioactivities. Alkaloids are widely reported to exhibit antimicrobial and diverse pharmacological activities, while flavonoids and phenols are well known for their antioxidant potential and ability to mitigate oxidative stress-related cellular damage. The presence of tannins suggests possible antimicrobial and wound-healing properties. In contrast, the fruit extract showed a different phytochemical distribution, with flavonoids, phenols, steroids, and terpenoids detected [Table S1, Supplementary Materials]. The absence of alkaloid and tannin compounds present in the leaf extract highlights organ-specific variations in secondary metabolite production. The detection of steroids exclusively in the fruit extract suggests potential anti-inflammatory or hormonal activities unique to the fruit. The shared presence of flavonoids, phenols, and terpenoids in both extracts indicates that both plant parts possess antioxidant capacity. These phytochemical differences emphasize the importance of plant part selection in future phytochemical and bioactivity investigations [7,8].

### 2.2. Total Phenolic Content (TPC) and Total Flavonoid Content (TFC)

The total phenolic and flavonoid contents of *P. cattleyanum* leaf and fruit extracts were evaluated to assess their potential bioactive properties. Based on an extract concentration of 10 mg/ml, the leaf extract exhibited significantly higher total phenolic content compared to the fruit extract under the conditions employed, indicating that leaves are a richer source of phenolic compounds [Tables S3 and S4 and Figure S2, Supplementary Materials]. Similarly, the total flavonoid content was markedly greater in the leaf extract than in the fruit extract based on comparative estimates rather than absolute flavonoid mass percentages within the crude extracts. The comparatively elevated TFC value observed for the leaf extract may partly reflect the strong colorimetric response of the concentrated dry extract, which exceeded the upper linear range of the catechin calibration curve under the assay conditions. The higher TFC value in leaf extract compared to the fruit extract may be partially attributed to the function of flavonoids in vegetative tissues. Leaves, which are directly exposed to environmental stressors such as UV radiation and herbivory, tend to accumulate more flavonoid compounds. These compounds function as protective secondary metabolites, contributing to photoprotection and antioxidant defense [9]. All values are expressed as mean ± SD [Tables S6 and S7 and Figure S4, Supplementary Materials]. These results suggest that the leaf extract exhibited a stronger response in phenolic and flavonoid colorimetric assays than the fruit extract, indicating comparatively greater abundance of antioxidant-associated constituents, consistent with previous studies on other plant species where phenolics and flavonoids contribute substantially to antioxidant activity. The pronounced difference between leaf and fruit extracts underscores the importance of plant part selection for functional ingredient development. The strong correlation observed in both gallic acid (R^2^ = 0.991) and catechin (R^2^ = 0.999) standard curves validates the reliability of the quantification methods employed. The calibration data and corresponding standard calibration curves are provided in Tables S2 and S5 and Figures S1 and S3 (Supplementary Materials).

The significantly higher TPC and TFC values observed in leaf extracts correlate well with the qualitative and GC–MS-based identification of phenolic compounds, flavonoids, and antioxidant terpenoids. Compounds such as caryophyllene, phytol, vitamin E, and β-caryophyllene, identified predominantly in leaf extracts, are well documented for their antioxidant potential. In contrast, the lower phenolic and flavonoid contents of fruit extracts are consistent with their comparatively weaker antioxidant activity.

When compared with previously reported values for *Psidium* species and other Myrtaceae plants, the TPC and TFC values obtained for *P. cattleyanum* leaf extracts are relatively high. Earlier studies on *Psidium guajava* and *P. cattleyanum* leaves have reported lower or comparable phenolic and flavonoid contents, depending on extraction method and plant part analyzed [10]. The elevated values observed in the present study may be attributed to the pooled multi-solvent extraction approach, which enhances the recovery of both polar and moderately non-polar antioxidant compounds.

One constraint of this study is the small number of replicates employed for some colorimetric tests. The TPC and TFC assessments were conducted with duplicate measurements, limiting the statistical strength and potentially not reflecting the full range of analytical variability. As a result, quantitative differences between samples should be viewed with caution, mainly as comparative trends. Future research that includes more biological and technical replicates would enhance the reliability and reproducibility of these findings.

### 2.3. Antioxidant Activity (DPPH Radical Scavenging Assay)

The results of the DPPH radical scavenging assay further elucidate the antioxidant potential of *Psidium cattleyanum* leaf and fruit extracts. The IC_50_ analysis revealed a significant disparity between the two plant parts: the leaf extract exhibited stronger DPPH radical scavenging activity under the experimental conditions employed with IC_50_ value of 47.10 ± 0.06 ppm, whereas the fruit extract demonstrated moderate antioxidant strength, with an IC_50_ value of 357.30 ± 0.64 ppm [Table S9, Supplementary Materials]. The IC_50_ value of ascorbic acid was found to be 23.90 ± 0.03 ppm [Table S8 and Figure S5, Supplementary Materials]. These findings are broadly consistent with the phytochemical characteristics observed for the extracts; however, direct mechanistic attribution cannot be established from the present data, which indicated that the leaves contained a more abundant array of phenolic, flavonoid, and terpenoid compounds strongly associated with free radical scavenging [9].

The antioxidant activity observed in the DPPH assay is likely associated with the combined effect of phenolic compounds, flavonoids, and antioxidant terpenoids. Phenolic compounds such as pyrogallol and flavonoids contribute directly to free radical scavenging, while terpenoids such as β-caryophyllene, phytol, and vitamin E may contribute to the antioxidant capacity, which are identified in GC–MS analysis (given in Section 2.5). Therefore, the observed results may reflect the cumulative effect of multiple phytochemicals present in the extracts.

**Figure 1:**
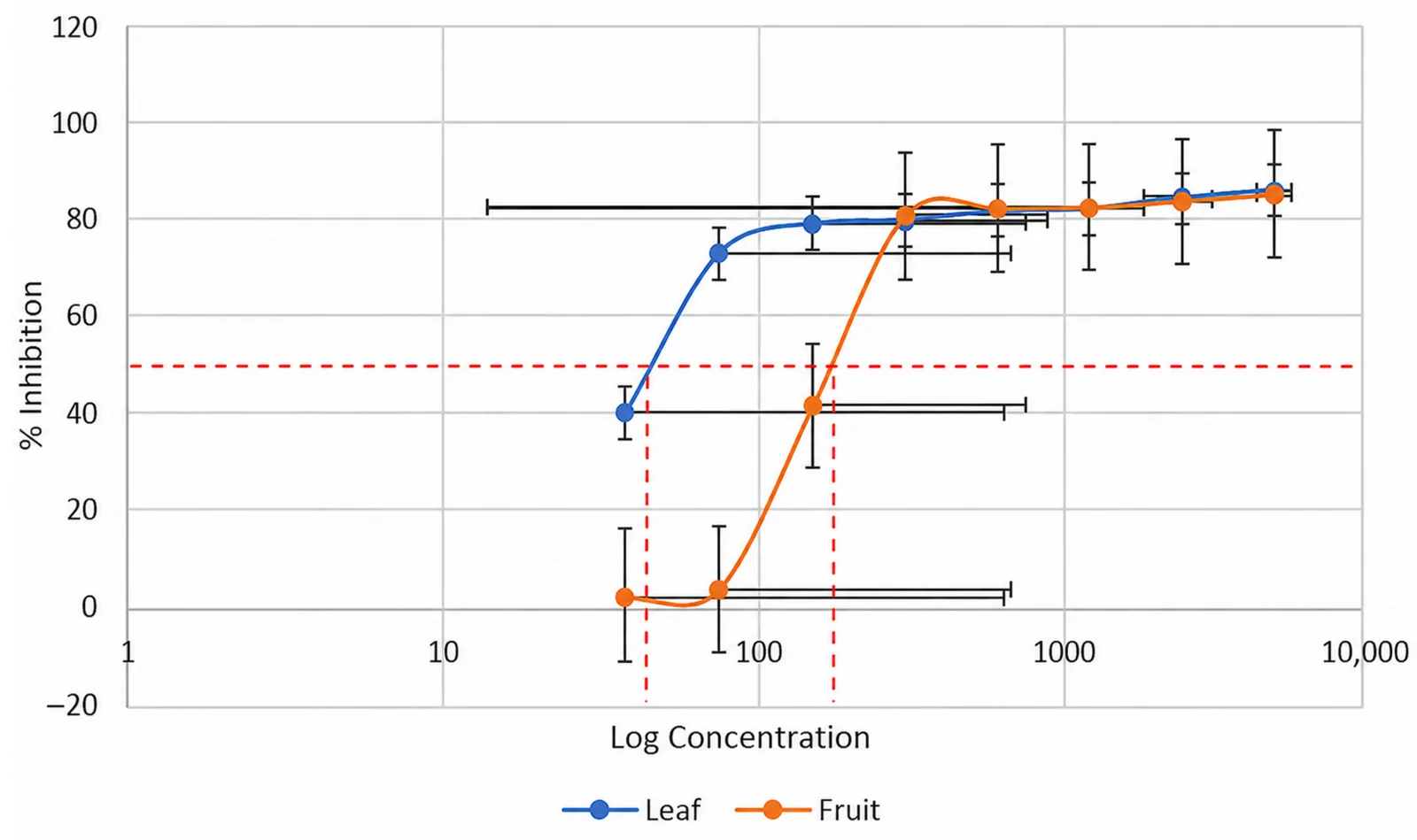
Dose–response relationship of leaf and fruit extracts showing the linear concentration range used for approximate IC_50_ estimation based on DPPH radical scavenging activity. The regression equation for leaf sample is y = 71.004x − 68.808 (R^2^ = 0.998), and for the fruit sample is y = 126.620x − 273.310 (R^2^ = 0.999). Dotted lines are IC_50_ values for fruit extract (357.30 ± 0.64 ppm) and leaf extract (47.10 ± 0.06 ppm). Data represent mean ± SD of two independent experiments (*n* = 2). IC_50_ values were estimated from linear interpolation of % inhibition ± SD at concentrations around 50% inhibition. The leaf extract exhibited higher total phenolic content and correspondingly lower IC_50_ values compared to the fruit extract, suggesting a possible contribution of phenolic compounds to the observed antioxidant activity. Error bars represent standard deviations calculated from duplicate independent measurements (*n* = 2). The linear relationship shown in Figure 1 corresponds to the linear region of the dose–response curve and is commonly employed for IC_50_ estimation in DPPH radical scavenging assays.

Although nonlinear regression using a four-parameter logistic model is generally preferred for IC_50_ determination, linear regression within the active inhibition range was used due to the limited number of concentration points available around the 50% inhibition region [Figure 1]. Consequently, the calculated IC_50_ values should be interpreted as approximate comparative indicators of antioxidant activity rather than definitive pharmacological parameters. Future studies employing a broader concentration range and nonlinear dose–response modeling would improve the accuracy of IC_50_ estimation.

The antioxidant activity assessment was based on a single chemical assay (DPPH), and IC_50_ values were estimated using linear regression rather than nonlinear dose–response modeling [Figure 1]. Therefore, the reported values should be interpreted as preliminary comparative measures of antioxidant activity.

### 2.4. Cytotoxicity Assessment by Brine Shrimp Lethality Bioassay

**Figure 2:**
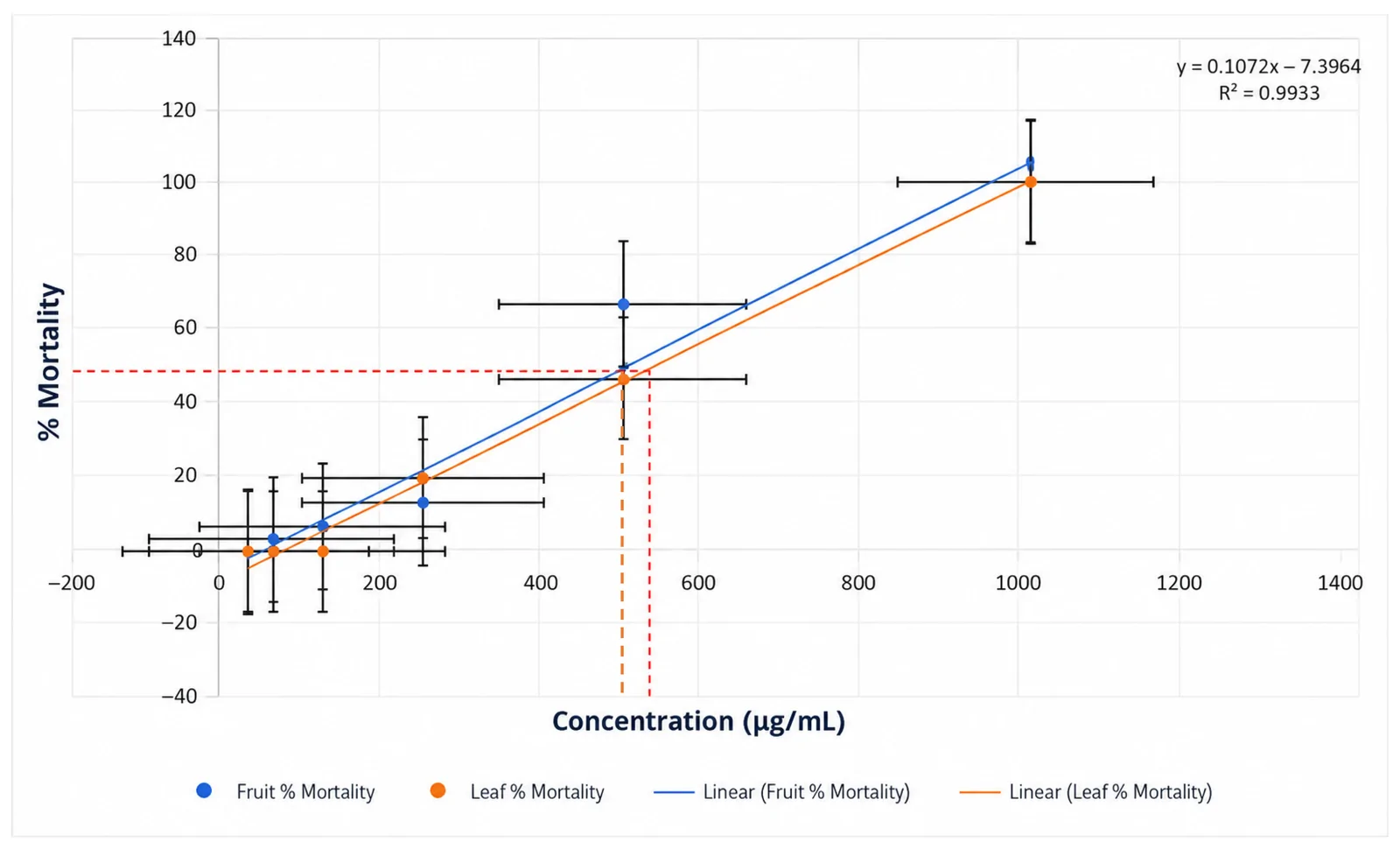
Concentration–mortality relationship of *Psidium cattleyanum* leaf and fruit extracts in the *Artemia salina* brine shrimp lethality assay. Dotted lines are LC_50_ values for fruit extract (402.60 ± 9.00 µg/mL) and leaf extract (522.14 ± 17.00 µg/mL). The regression equation is y = 0.1072x − 7.3964. Data represent mean ± SD of three replicates (*n* = 3). LC_50_ value for fruit extract was calculated using linear interpolation of % mortality ± SD at concentrations surrounding 50% mortality.

Cytotoxicity testing using the *Artemia salina* brine shrimp assay indicated that both extracts fall within acceptable biological safety ranges [Figure 2]. The fruit extract exhibited an LC_50_ of 402.60 ± 9.00 µg/mL, indicating moderate cytotoxicity, while the leaf extract exhibited a slightly lower effect with an LC_50_ of 522.14 ± 17.00 µg/mL, classifying it as mildly cytotoxic [Table S10, Supplementary Materials]. The positive control potassium dichromate showed an LC_50_ value of 7.96 ± 0.68 µg/mL [probit analysis was performed—Table S11 and Figure S6, Supplementary Materials]. These cytotoxicity levels are typical of plant extracts containing bioactive secondary metabolites and indicate mild cytotoxic potential consistent with the presence of bioactive secondary metabolites. Collectively, the IC_50_ and LC_50_ values indicate differences in antioxidant and cytotoxic responses between leaf and fruit extracts. These findings provide preliminary evidence of biological activity and support further phytochemical and mechanistic investigations [11].

### 2.5. GC–MS Profiling of Leaf and Fruit Extracts

**Figure 3:**
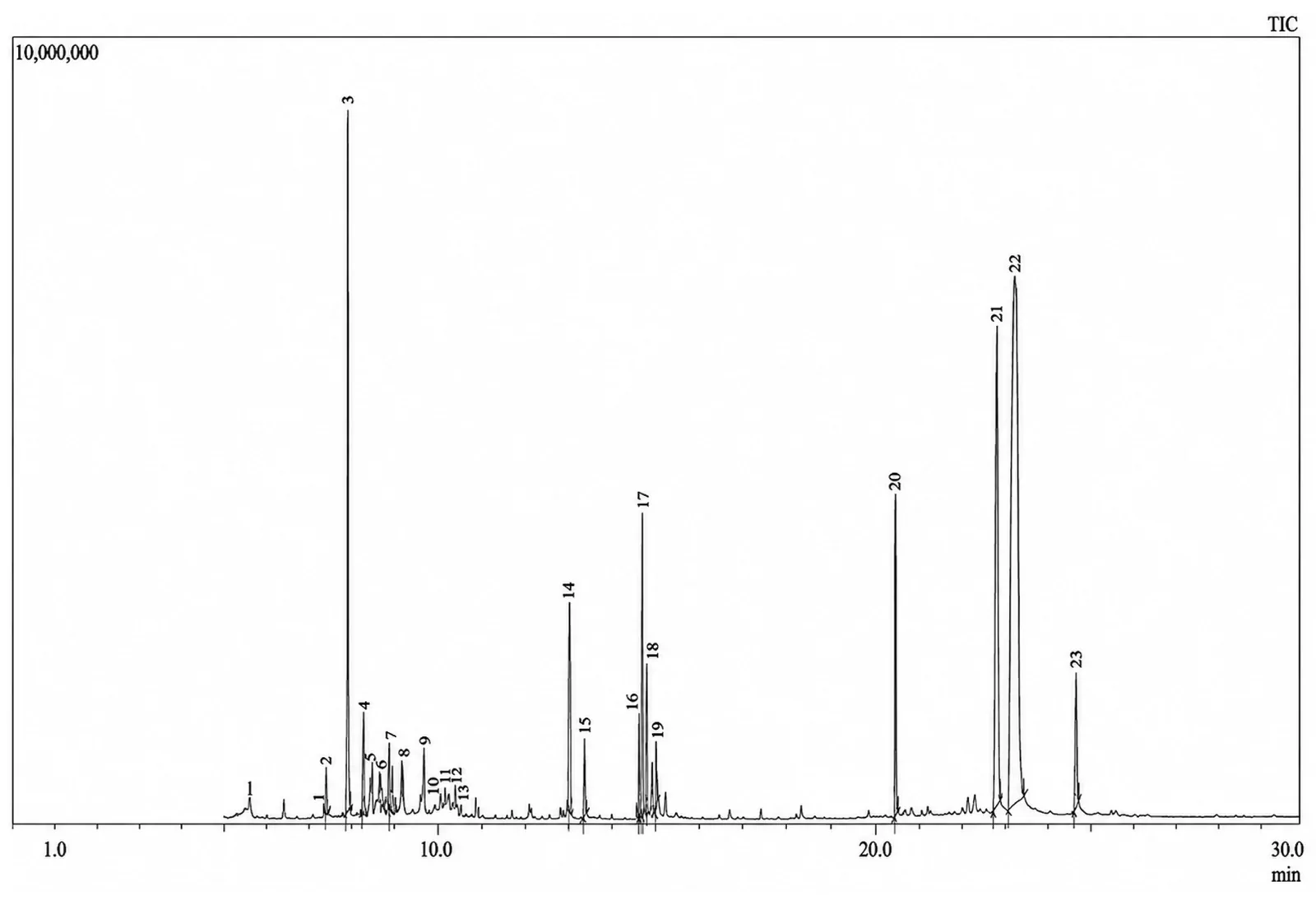
GC–MS chromatogram of *P. cattleyanum* leaf extract. The retention times indicated above the chromatographic peaks correspond to the compounds listed with retention times in Table S12 in Supplementary Material.

**Figure 4:**
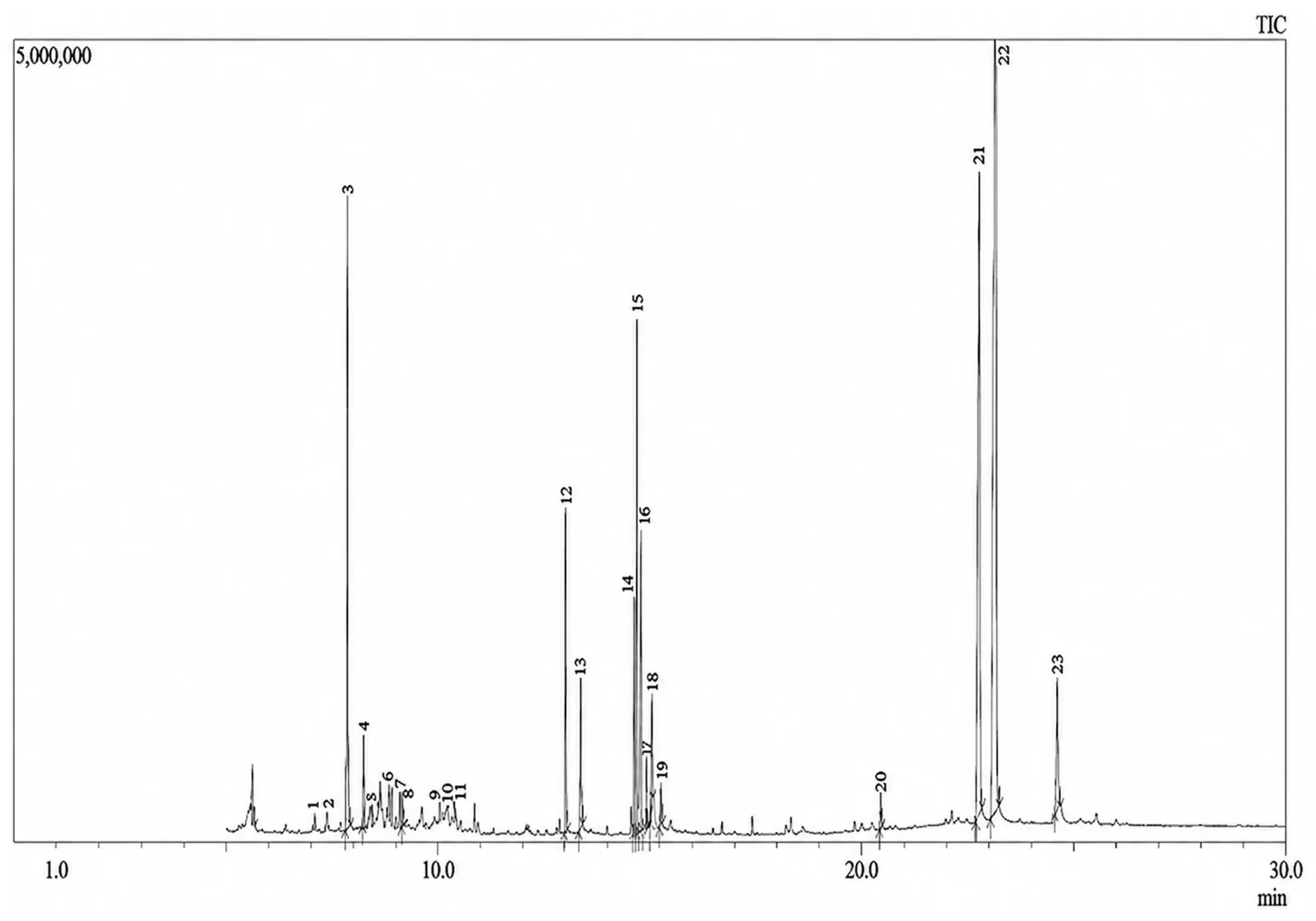
GC–MS chromatogram of *P. cattleyanum* fruit extract. The numbers indicated above the chromatographic peaks correspond to the compounds listed with retention times in Table S13 in Supplementary Materials.

Gas chromatography–mass spectrometry (GC–MS) analysis further highlighted the chemical distinctiveness of the leaf and fruit extracts (Figure 3 and Figure 4; Tables S12 and S13). Although several compounds identified in the extracts have previously been reported to exhibit antioxidant, anti-inflammatory, antimicrobial, or cytotoxic activities, direct attribution of the observed biological effects to individual constituents is not possible based on the present data. Biological activity is likely influenced by the combined action of multiple compounds, together with factors such as concentration, bioavailability, and synergistic or antagonistic interactions. Therefore, the associations discussed below should be regarded as potential relationships rather than confirmed mechanisms. Major phytochemical constituents in the pooled extracts of *Psidium cattleyanum* were tentatively identified based on mass spectral matching with the NIST Mass Spectral Library and Wiley Mass Spectral Library [12,13,14], with comparison of experimentally calculated retention indices (RI) with literature values obtained for non-polar columns. This combined approach enhances the reliability of compound annotation and corresponds to Schymanski confidence level 2 (probable structure identification). Retention indices were determined using a homologous series of n-alkane standards (C10–C33) analyzed under identical chromatographic conditions (Supplementary Materials Table S15, Figure S7). Most compounds showed acceptable agreement with literature values, with RI deviations generally within 20 units, supporting the validity of the proposed identifications. Nevertheless, definitive structural confirmation would require analysis using authentic reference standards. The majority of compounds identified, including terpenoids, fatty acids, sterols, and phenolic derivatives, are consistent with those reported in *Psidium* species and other members of the Myrtaceae family. These compound classes are characteristic of aromatic and medicinal plants and are widely associated with antioxidant and other pharmacological activities [4,5]. Detailed compound profiles are provided in Tables S12 and S13 (Supplementary Materials). The present discussion focuses on compounds detected at relatively higher abundance and those with reported biological relevance to the observed antioxidant and cytotoxic activities. Compounds suspected to arise from contaminants, solvents, or library over-matching were excluded from mechanistic interpretation to avoid overestimation of their biological significance.

#### 2.5.1. Leaf Extract

The GC–MS analysis of the *Psidium cattleyanum* leaf extract revealed a phytochemical profile dominated by terpenoid constituents, particularly sesquiterpenes, together with tocopherol and triterpenoid-related compounds. Among the identified compounds, caryophyllene (Peak 3) was the most abundant phytochemical constituent excluding the internal standard, accounting for 13.42% of the total chromatographic peak area. dl-α-Tocopherol was the second major constituent (6.89%), followed by squalene (3.96%), 6-octadecenoic acid methyl ester (3.84%), γ-sitosterol (2.98%), hexadecanoic acid methyl ester (2.51%), phytol (2.08%), and β-cadinene (1.43%). The predominance of sesquiterpenoid constituents, together with the presence of tocopherol and other bioactive metabolites, suggests a chemically complex extract enriched with antioxidant potential.

Sesquiterpenes represented a prominent chemical class in the leaf extract. Caryophyllene and humulene were detected at relative abundances of 13.42% and 1.50%, respectively, while lower levels of such sesquiterpenes as ylangene (0.17%), γ-muurolene (0.59%), α-maaliene (0.63%), caryophyllene oxide (0.90%), selina-6-en-4-ol (0.21%), τ-cadinol (0.30%), α-cadinol (0.43%), copaene (0.75%), α-selinene (0.81%), and β-cadinene (1.43%) were also observed.

Caryophyllene and humulene are widely reported to possess antioxidant, anti-inflammatory, and cytoprotective activities [14,15,16,17], whereas caryophyllene oxide has additionally been associated with antifungal activity [15,16,17]. The comparatively high abundance of caryophyllene suggests that sesquiterpenoid constituents may be among several compound classes potentially associated with the observed biological responses of the leaf extract. Particularly, β-caryophyllene has been identified as a selective CB2 receptor agonist and is associated with anti-inflammatory effects without central nervous system involvement [18,19].

Ylangene possesses antimicrobial, antioxidant, and anti-inflammatory properties. γ-Muurolene is recognized for its antibacterial, antifungal, insecticidal, and antioxidant activities. β-Cadinene demonstrates antimicrobial, anti-inflammatory, and antioxidant effects. α-Selinene is characterized by its anti-inflammatory, antioxidant, and antimicrobial properties. τ-Cadinol has been found to exhibit antifungal, antibacterial, and cytotoxic effects against certain cancer cell lines. α-Cadinol is noted for its strong antimicrobial, antifungal, antioxidant, and anti-inflammatory activities. Selina-6-en-4-ol is known for its antioxidant and antimicrobial activities, particularly in essential oils [15].

Fatty acid derivatives detected in the leaf extract included hexadecanoic acid methyl ester (2.51%), n-hexadecanoic acid (1.04%), 6-octadecenoic acid methyl ester (3.84%), methyl stearate (0.63%), and 9-octadecenoic acid (1.04%). Among these, 6-octadecenoic acid methyl ester was the predominant lipid-derived constituent. Fatty acid esters and related lipid compounds have been reported to exhibit antioxidant, antimicrobial, and membrane-protective properties [20], potentially contributing to the overall bioactivity of the extract.

Phytol (2.08%) and squalene (3.96%) were also identified as notable constituents of the leaf extract. Phytol is a diterpene alcohol recognized as a precursor in the biosynthesis of vitamins E and K and has been associated with antioxidant and antimicrobial activities [21]. Squalene, an important triterpene intermediate in sterol biosynthesis, is widely recognized for its antioxidant and protective roles against oxidative stress. In addition, the high abundance of dl-α-tocopherol (6.89%) further supports the strong antioxidant potential of the leaf extract. Tocopherols are well-established lipid-soluble antioxidants. However, their specific contribution to the observed assay responses cannot be determined from the present study.

The phytosterol γ-sitosterol was detected at a relative abundance of 2.98%. Phytosterols are structurally analogous to cholesterol and are associated with anti-inflammatory, hypocholesterolemic, and membrane-stabilizing effects [22,23]. The presence of γ-sitosterol alongside tocopherols and terpenoid constituents further supports the presence of phytochemicals that have been reported to exhibit biological activities in previous studies.

Overall, the phytochemical profile of the leaf extract is dominated by sesquiterpenes and terpenoids together with some lipid-derived compounds, along with less phenolic constituents, indicating notable biological potential. The detected compounds are associated with antioxidant, anti-inflammatory, and antimicrobial activities; however, these functional attributes are inferred from previously reported studies on individual compounds. Further bioassay-guided fractionation and mechanistic investigations are required to validate their biological effects.

#### 2.5.2. Fruit Extract

The GC–MS analysis of *Psidium cattleyanum* fruit extract revealed a phytochemical composition characterized predominantly by unsaturated fatty acid derivatives and other lipid-related constituents, accompanied by sesquiterpenoid compounds and dl-α-tocopherol. Excluding the internal standard, caryophyllene (Peak 3) was the major constituent, accounting for 10.18% relative abundance. dl-α-Tocopherol (7.35%) and 8,11,14-eicosatrienoic acid methyl ester (7.21%) were also present at comparatively high levels, followed by phytol (4.75%), hexadecanoic acid methyl ester (4.34%), 9,12-octadecadienoic acid methyl ester (3.32%), γ-sitosterol (3.67%), 9-octadecenoic acid (2.27%), and n-hexadecanoic acid (2.66%). The predominance of unsaturated fatty acid derivatives along with tocopherol suggests a lipid-rich phytochemical profile with potential antioxidant and cytoprotective relevance.

Among the detected compounds, eugenol was identified as a minor phenolic constituent, accounting for 0.17% relative abundance. It is widely recognized for its antioxidant, antimicrobial, and anti-inflammatory activities and may contribute, at least partially, to the observed bioactivity of the fruit extract [24]. α-Copaene was also detected at relatively low abundance (0.29%). This sesquiterpene hydrocarbon, commonly reported in essential oils, has been associated with antimicrobial and insecticidal properties [25]. Although present at low levels, the occurrence of these compounds represents potential contribution to the overall phytochemical profile, although their individual roles in the observed biological responses remain uncertain.

Sesquiterpenes identified in the fruit extract include caryophyllene (10.18%), humulene (1.49%), and caryophyllene oxide (0.16%), all of which were also detected in the leaf extract. Caryophyllene is among the dominant constituents of the fruit extract, whereas humulene and caryophyllene oxide are present in comparatively lower abundance. These terpenoids are widely associated with antioxidant, anti-inflammatory, and antimicrobial activities, potentially contributing to the observed bioactivity of the extract. The fruit sample of *Psidium cattleyanum* contains several sesquiterpenes that collectively contribute to its medicinal and ecological properties. These include γ-muurolene (0.34%), a sesquiterpene hydrocarbon known for its antimicrobial, antioxidant, anti-inflammatory, and insecticidal activities, which supports the plant’s defense mechanisms; cis-calamenene (0.80%), another sesquiterpene hydrocarbon with strong antimicrobial, antioxidant, anti-inflammatory, insecticidal, and reported anticancer effects; and α-maaliene (0.55%), which enhances the fruit’s aroma while providing antimicrobial, antioxidant, and anti-inflammatory benefits. Oxygenated sesquiterpenes such as cubenol (0.49%), τ-cadinol (0.73%), and τ-muurolol (0.57%) further enhance the essential oil’s bioactivity, each exhibiting broad-spectrum antibacterial, antifungal, antioxidant, anti-inflammatory, and insecticidal properties, with τ-cadinol also demonstrating cytotoxic activity against certain cancer cell lines. Collectively, these sesquiterpenes reinforce the essential oil’s diverse bioactivity, supporting both the plant’s defense system and its potential pharmacological applications [15].

Several fatty acid derivatives were identified in the fruit extract, including hexadecanoic acid methyl ester (4.34%), n-hexadecanoic acid (2.66%), 9,12-octadecadienoic acid methyl ester (3.32%), 8,11,14-eicosatrienoic acid methyl ester (7.21%), 9-octadecenoic acid (2.27%), methyl stearate (0.98%), and octadecanoic acid (0.75%). Among these, 8,11,14-eicosatrienoic acid methyl ester represented the predominant lipid-derived constituent. Unsaturated fatty acid esters have been reported to exhibit antioxidant, anti-inflammatory, and membrane-modulating activities [26,27], suggesting that these compounds may contribute to the biological effects observed for the fruit extract.

Phytol (4.75%), dl-α-tocopherol (7.35%), and γ-sitosterol (3.67%) were also detected at notable levels in the fruit extract. The comparatively high abundance of dl-α-tocopherol suggests that tocopherol-related compounds constitute a major antioxidant component of the fruit extract. Tocopherols are well-established lipid-soluble antioxidants capable of protecting cellular membranes from oxidative damage, while phytol and phytosterols have additionally been associated with antioxidant, antimicrobial, and protective biological effects. The occurrence of these constituents in both leaf and fruit extracts further supports their contribution to the overall bioactivity of the plant.

Overall, the phytochemical composition of the fruit extract reflects a mixture of sesquiterpenoid, lipid-derived, and tocopherol-related compounds associated with antioxidant, anti-inflammatory, and antimicrobial properties. The antioxidant activity may be partly associated with phenolic- and flavonoid-related compounds suggested by phytochemical screening, while GC–MS primarily reflects volatile and semi-volatile constituents detectable under the applied analytical conditions of non-derivatized extracts. However, these functional properties are inferred from previously reported activities of individual compounds. Further bioassay-guided fractionation, toxicity evaluation, and mechanistic studies are required to validate their biological effects.

Comparative GC–MS profiling of *Psidium cattleyanum* leaf and fruit extracts revealed both shared and tissue-specific phytochemical characteristics. Several constituents, including caryophyllene, humulene, phytol, dl-α-tocopherol, squalene, and γ-sitosterol, were detected in both extracts, indicating conserved sesquiterpenoid and lipid-related biosynthetic pathways across plant tissues. However, substantial quantitative differences were observed in their relative abundances.

The leaf extract was characterized by a comparatively higher abundance of sesquiterpenoid constituents, particularly caryophyllene, together with relatively higher levels of squalene and other sesquiterpene hydrocarbons. In contrast, the fruit extract exhibited a greater contribution of unsaturated fatty acid-derived compounds, including 8,11,14-eicosatrienoic acid methyl ester and 9,12-octadecadienoic acid methyl ester, together with dl-α-tocopherol. During evaluation of the internal standard, a peak corresponding to dl-α-tocopherol was detected in the α-tocopheryl acetate solution, indicating that a possible artefactual contribution under GC–MS conditions cannot be completely excluded. However, dl-α-tocopherol was also detected in the original unspiked plant extracts, suggesting a plant-associated contribution. Therefore, the detected dl-α-tocopherol signal may represent a combination of naturally occurring compound and potential analytical contribution, and the GC–MS results are interpreted as semi-quantitative profiles rather than absolute quantification. Accordingly, all relative abundance and semi-quantitative concentration values reported for dl-α-tocopherol were corrected by considering the contribution of the internal standard prior to data interpretation.

Hexadecanoic acid methyl ester detected in the methanol extract may originate from methylation of naturally occurring fatty acids during methanolic extraction, which is a common phenomenon in GC–MS analysis. These compositional differences indicate tissue-specific metabolic specialization within the plant.

Notably, caryophyllene was the predominant constituent in both extracts but occurred at substantially higher abundance in the leaf extract than in the fruit extract, suggesting greater accumulation of sesquiterpenoid constituents in leaf tissues. Similarly, phytol and unsaturated fatty acid esters were comparatively more abundant in the fruit extract, whereas sesquiterpenoid hydrocarbons and squalene were relatively enriched in the leaf extract.

Both extracts contained phytosterols and fatty acid derivatives associated with membrane structure, plant defense, and reported pharmacological activities such as antioxidant, anti-inflammatory, and hypocholesterolemic effects. Nevertheless, the biological functions discussed herein are inferred from previously reported studies on individual compounds, and direct mechanistic attribution cannot be established solely from GC–MS profiling.

Certain low-abundance compounds and atypical spectral matches were interpreted cautiously, as they may represent analytical artifacts, environmental contaminants, or spectral library mismatches. Such constituents were therefore excluded from mechanistic interpretation to maintain analytical rigor.

Overall, the comparative phytochemical profiles demonstrate that the leaf extract is relatively enriched in sesquiterpenoid constituents, whereas the fruit extract is characterized by higher levels of lipid-derived metabolites, including unsaturated fatty acid derivatives and dl-α-tocopherol. The observed differences in phytochemical composition broadly correspond to differences in assay responses between the extracts; however, direct mechanistic relationships cannot be established without targeted isolation and bioactivity-guided investigations.

The identified compounds should be considered tentatively assigned based on GC–MS spectral matching and RI comparison, as definitive structural elucidation would require confirmation using authentic standards and complementary spectroscopic techniques. Several peaks remained unidentified due to the absence of reliable spectral and RI matches, emphasizing the chemical complexity of extracts. Combining phytochemical profiling with bioactivity assays offers a valuable initial framework for identifying compound classes linked to biological activity. However, to establish definitive structure–activity relationships, it is essential to isolate compounds, conduct quantitative analyses, and validate mechanisms.

In this study, GC–MS analysis was conducted on pooled extracts without derivatization, which restricts the detection of highly polar and thermally unstable phytochemicals, such as numerous phenolic acids and flavonoids. Although direct GC–MS analysis of non-derivatized extracts enabled the characterization of volatile and semi-volatile constituents, derivatization approaches such as BSTFA silylation would be expected to improve the detection of highly polar metabolites including phenolic acids, flavonoids, sugars, and glycosylated compounds, thereby providing broader metabolite coverage. Such analyses were beyond the scope of the current work and may be considered in future studies. Therefore, the present GC–MS results should not be interpreted as an exhaustive metabolomic characterization of the extracts. Additionally, while pooling sequential solvent extracts broadens metabolite coverage, it reduces the chemical resolution specific to each solvent. Future research should consider using derivatization-based GC–MS and LC–MS/MS for more thorough metabolite profiling.

The biological assays utilized in this research serve as preliminary screening methods and are not indicative of therapeutic effectiveness, safety, or pharmacological behavior in living organisms. As a result, the biological significance of the activities observed needs to be validated through specific cellular, mechanistic, and animal investigations. Additionally, this study identifies links between phytochemical composition and responses in biological assays but does not demonstrate causal relationships. Therefore, the biological activities attributed to individual compounds should be considered with caution until they are verified through bioassay-guided fractionation, quantitative evaluations, and mechanistic research.

## 3. Materials and Methods

### 3.1. Collection of Samples

Mature fruits and fresh leaves of *Psidium cattleyanum* (Myrtaceae) were collected from healthy plants in Kandy, Central province of Sri Lanka, at an altitude of approximately 560 m above sea level. Samples were collected during the fruiting season (July–November), and this location was selected based on to the abundance, accessibility, and wide distribution of the species. The sampled plants were part of a connected population within the same ecological area. Fruits were selected based on uniform red coloration, firmness, and absence of physical damage or disease symptoms. Leaves were carefully collected to minimize damage and ensure sample quality. Approximately 1 kg of leaves and 1 kg of fruits (approximately 25) were collected. To create a representative composite sample and reduce the impact of variation between plants on the overall phytochemical profile, plant materials from five separate plants were combined and then sub-sampled to produce three analytical replicates. This method does not allow for the assessment of biological variability among individual plants. Future research should include independent biological replicates from various plants and locations to more accurately evaluate natural phytochemical differences. Leaves and fruits were placed in separate sterile sampling bags, labeled with the date, location, and species information, and refrigerated at 4.0 °C to preserve sample integrity. The frozen samples were maintained at a consistent temperature until further processing and analysis [28].

### 3.2. Plant Extraction Method

A mass of approximately 5.00 g of dried leaf and fruit powders was subjected to a continuous sequential solvent extraction (solvent-to-sample ratio: 5:1) using 25.00 mL volumes for each hexane, dichloromethane (DCM), and methanol. First, the grinded plant material was mixed with hexane and sonicated for enhanced penetration of non-polar solvents. The solvent was decanted, followed by addition of DCM and sonication. Finally, methanol was added and the mixture was sonicated again. Ultrasonic-assisted sonication was carried out at 40 °C with 15 mins time intervals in a medium power ultrasonic bath.

The combined multi-solvent extraction process was continued over 5 days, with intermittent shaking and sonication to maximize extraction efficiency. Following sequential extraction, all solvent fractions were pooled prior to solvent removal in order to obtain a comprehensive extract representative of the total metabolite profile, rather than solvent-specific fractions. The extracts were concentrated under reduced pressure using a rotary evaporator at 45.0–50.0 °C. The dried residue was re-dissolved in 10.00 mL of methanol, transferred into sealed vials, refrigerated at 4.0 °C and analyzed within two weeks to minimize degradation [29,30]. The sequential extraction using hexane, dichloromethane, and methanol was employed to maximize the recovery of metabolites with varying polarity. The objective of the extraction procedure was to obtain a broad-spectrum phytochemical from plant materials [31]. Following solvent removal, extracts were reconstituted in methanol to obtain a homogeneous solution suitable for analytical handling. While methanol may not dissolve all non-polar constituents with equal efficiency, this approach is commonly applied for GC–MS compatibility and ability to solubilize a broad range of phytochemical classes. Consequently, GC–MS results are interpreted as indicative rather than exhaustive profiles of volatile and semi-volatile constituents.

### 3.3. Phytochemical Screening

For alkaloids, mixing 2.00 mL of plant extract with 0.20 mL of dilute hydrochloric acid (1–2 M HCl) and 1.00 mL of Mayer’s reagent resulted in a yellowish color or creamy white precipitate, indicating the presence of alkaloids. Flavonoids were detected by adding five drops of concentrated hydrochloric acid (~37% *w*/*v* HCl) and magnesium turnings to a small amount of the extract, with immediate red color development signifying their presence. Tannins were identified by mixing 5.00 mL of the extract with 2.00 mL of 5% (*w*/*v*) ferric chloride (FeCl_3_) solution, resulting in a greenish-black precipitate. Saponins were detected by shaking 0.50 g of the ground plant sample with 5.00 mL of distilled water and warming gently, with persistent frothing indicating their presence. Terpenoids were identified by adding 1.00 mL of acetic anhydride and three drops of concentrated sulfuric acid (H_2_SO_4_) to 2.00 mL of the extract, with pink, red, magenta, or violet color appearing after 5 min. Phenols were detected by mixing 1.00 mL of the extract with 1.00 mL of water and 1–2 drops of ferric chloride (FeCl_3_) solution (5% *w*/*v*), resulting in blue, green, red, or purple colors. Sterols and triterpenes were identified by adding 2.00 mL of acetic anhydride and 2.00 mL of concentrated sulfuric acid to 5.00 mL of the extract, with a color change from violet to blue confirming the presence of steroids. Carbohydrates were detected by treating the extract with Molisch reagent and slowly adding concentrated sulfuric acid, resulting in a purple-violet ring at the junction. These tests allow for rapid qualitative screening of various phytochemical compounds in plant extracts [32,33,34].

### 3.4. Determination of Total Phenolic and Flavonoid Contents

#### 3.4.1. Total Phenolic Content (TPC)

Total phenolic content was determined using the Folin–Ciocalteu (FC) method [see calibration curve in Figure S1, Supplementary Materials]; with slight modifications. Briefly, 0.50 mL of methanolic extract or standard solution was mixed with 3.00 mL of distilled water, 1.00 mL of FC reagent, and incubated for 5–8 min in the dark. Subsequently, 2.00 mL of saturated sodium carbonate solution was added, and the volume was adjusted to 10.00 mL with distilled water. After incubation for 30 min in dark conditions, absorbance was measured in duplicates at 765 nm using a Thermo scientific ^TM^ Evolution 201 UV–Vis spectrophotometer (Thermo Fisher Scientific, Waltham, MA, USA).

Gallic acid was used as the standard, and a calibration curve was constructed over the concentration range of 10–320 µg/mL. Calibration curves were constructed using the final concentrations of the standards present in the reaction mixture after completion of the colorimetric assay.

Calculations were done using the equation;

TPC=(C×V)/m

where

- C = gallic acid concentration obtained from the calibration curve (µg/mL)
- V = final volume of the reaction mixture (mL)
- m = mass of dry extract used in the reaction (g)

TPC of the samples was calculated from this equation and expressed as mg gallic acid equivalents (GAE) per gram of dry extract (mg GAE/g dry extract) with standard deviation [35].

All measurements were performed in duplicate, and results are expressed as mean ± standard deviation. The duplicate measurements were used to provide preliminary quantitative estimates; however, the limited replication should be considered when interpreting quantitative differences between samples. Due to the limited number of replicates, formal inferential statistical comparisons between extracts were not performed. Results are presented as mean ± standard deviation and interpreted descriptively.

#### 3.4.2. Total Flavonoid Content (TFC)

Total flavonoid content was determined using the aluminum chloride colorimetric method [see calibration curve in Figure S1, Supplementary Materials]. Methanolic extract (0.50 mL of diluted extract) was mixed with 3.00 mL of distilled water and 0.30 mL of 5% sodium nitrite, allowed to react for 5 min. Subsequently, 0.30 mL 10% aluminum chloride was added and incubated for 6 min, followed by 2.00 mL 1 M sodium hydroxide. The volume was adjusted to 10.00 mL with distilled water, and the mixture was kept in the dark for 15 min at room temperature. Absorbance was measured in duplicates at 510 nm using Thermo scientific ^TM^ Evolution 201 UV–Vis spectrophotometer.

Catechin was used as the reference standard, and a calibration curve was prepared in the concentration range of 10–320 µg/mL. Calibration curves were constructed using the final concentrations of the standards present in the reaction mixture after completion of the colorimetric assay.

Calculations were done using the equation;

TFC=(C×V)/m

where

- C = catechin equivalent concentration obtained from the calibration curve (µg/mL)
- V = final volume of the extract solution used for the assay (mL)
- m = mass of dry extract (g) corresponding to the aliquot used in the reaction mixture

TFC of the samples was calculated from this equation and expressed as mg catechin equivalents (CE) per gram of dry extract (mg CE/g dry extract) [36].

All measurements were performed in duplicate, and results are expressed as mean ± standard deviation. Standard deviation was calculated from replicate absorbance measurements and propagated through the final concentration calculations. The duplicate measurements were used to provide preliminary quantitative estimates; however, the limited replication should be considered when interpreting quantitative differences between samples. Due to the limited number of replicates, formal inferential statistical comparisons between extracts were not performed. Results are presented as mean ± standard deviation and interpreted descriptively.

### 3.5. The DPPH RSA (Radical Scavenging Activity)

The antioxidant activity of the extracts was assessed utilizing the DPPH (2,2-diphenyl-1-picrylhydrazyl) free radical scavenging assay. A 0.01% (*w/v*) DPPH solution was freshly prepared by dissolving DPPH in methanol and was stored protected from light. Extract stock solutions (10,000 ppm) were prepared in methanol and subsequently serially diluted to achieve the desired concentration range (10,000–78 ppm). For each assay, 2.00 mL of the DPPH solution was combined with 1.00 mL of the plant extract solution. The reaction mixtures were incubated in complete darkness for 30 min. Absorbance was measured in triplicates independent experiments at 517 nm using a Thermo scientific ^TM^ Evolution 201 UV–Visible spectrophotometer.

The radical scavenging activity was calculated using the following formula:

$$RSA\;(\%)=[(A_{0}-A_{s})/A_{0}]\times 100$$

where A_0_ is the absorbance of the control and A_s_ is the absorbance of the sample. All measurements were performed in triplicate, and results are expressed as mean ± standard deviation.

Ascorbic acid was employed as a standard antioxidant (5–1000 ppm). IC_50_ values for ascorbic acid and plant extracts were estimated using linear regression analysis of log_10_-transformed concentrations versus percentage inhibition within the linear region of the dose–response curve [37].

### 3.6. Brine Shrimp Lethality Bioassay (BSLA)

The cytotoxicity of the plant extracts was evaluated utilizing the brine shrimp lethality bioassay (BSLA). Approximately 1.00 g of *Artemia salina* (Brine shrimp) eggs was introduced into artificial seawater and incubated under gentle aeration for 48 h. The hatched nauplii were collected from the illuminated area using a Pasteur pipette. Stock solutions of the extracts were prepared in seawater and serially diluted to achieve final concentrations of 31.25, 62.5, 125, 250, 500, and 1000 µg/mL. For each concentration, 10 nauplii were transferred into wells containing 2.00 mL of the test solution. The plates were maintained at room temperature under gentle light for 24 h.

Following incubation, the surviving and deceased nauplii were counted in triplicates, and the percentage mortality was calculated by the equation;

$$Mortality\;(\%)=[(N_{0}-N_{t})/N_{0}]\times 100$$

where N_0_ is the number of larvae in the control group and N_t_ is the number of surviving larvae after exposure.

LC_50_ values were determined using probit analysis and log-dose mortality plots [38].

### 3.7. GC–MS Analysis

GC–MS analysis was performed using a Shimadzu GC–MS QP2010 system (Shimadzu Corporation, Kyoto, Japan). The gas chromatograph was equipped with a non-polar Rtx-5Sil MS capillary column (30 m × 0.25 mm i.d., 0.25 µm film thickness). Helium was used as the carrier gas at a constant flow rate of 1.69 mL/min. The oven temperature was initially set at 50 °C and programmed to increase to 300 °C at a rate of 10 °C/min, followed by an isothermal hold for 38.5 min to ensure complete elution of late-retaining compounds and column conditioning between runs.

Samples were injected using the instrument autosampler and the injector was operated in splitless mode, with injector, ion source, and interface temperatures maintained at 250 °C, 200 °C, and 250 °C, respectively. Mass spectra were acquired in electron ionization (EI) mode at an ionization energy of 70 eV over a mass scan range of *m*/*z* 50–500. A solvent delay of approximately 5 min was applied to minimize solvent interference [39].

An aliquot (1 µL) of each methanolic extract was injected for GC–MS analysis. The dry residue concentrations of the extracts prior to analysis were 95.8 mg/mL for the leaf extract and 74.2 mg/mL for the fruit extract. For semi-quantitative analysis, α-tocopheryl acetate was used as an internal standard (IS). A stock solution of α-tocopheryl acetate (10 ppm) was prepared, and 500 µL of this solution was added to 1 mL of each extract prior to GC–MS analysis. During evaluation of the internal standard, a dl-α-tocopherol peak was observed in the α-tocopheryl acetate solution under the employed GC–MS conditions. As a result, the possibility of an analytical artefact associated with dl-α-tocopherol formation from the internal standard during GC–MS analysis was considered. The detected dl-α-tocopherol signals were interpreted cautiously, and the corresponding results were interpreted as semi-quantitative estimates. Semi-quantification estimates of identified compounds were performed based on peak area normalization relative to the internal standard and are intended solely as comparative estimates of compound abundance rather than absolute concentrations. The direct GC–MS method was optimized for the analysis of natural volatile and semi-volatile constituents. Therefore, no chemical derivatization (e.g., BSTFA silylation) was applied.

Semi-quantitative estimates of detected compounds were obtained based on relative peak area normalization using an internal standard to ensure run-to-run consistency. The total chromatographic run time for each sample was 63.5 min, consisting of a 25 min temperature ramp followed by a 38.5 min final isothermal hold [Table S14, Supplementary Materials]. The chromatographic conditions were based on the instrument-recommended method provided by the manufacturer and were applied consistently throughout the study. Although alternative temperature programs with shorter final hold times may improve analytical efficiency, the selected conditions ensured complete elution of late-retaining constituents and consistency across all analyses. Relative peak area percentages together with internal standard normalization were used for semi-quantitative estimation of compound abundance within each chromatogram. This approach provides comparative semi-quantitative information suitable for phytochemical profiling under identical analytical conditions. These values represent relative semi-quantitative estimates based on relative peak area percentages. Phytochemicals were tentatively identified by comparison of mass spectra with the National Institute of Standards and Technology (NIST) Mass Spectral Library together with retention index (RI) comparison against literature data [40].

A homologous series of n-alkanes (C10–C33) was analyzed under identical chromatographic conditions following the instrument-recommended method provided by the manufacturer (Shimadzu Corporation) and applied consistently for all sample analyses.

Retention indices (RI) were calculated using the Van den Dool and Kratz equation for temperature-programmed gas chromatography:

$$RI=100n+100\times [(t_{r}(x)-t_{r}(n))/(t_{r}(n+1)-t_{r}(n))]$$

where t_r_(x) is the retention time of the target compound, t_r_(n) and t_r_(n + 1) are the retention times of the n-alkanes eluting immediately before and after the compound, and n is the carbon number of the preceding n-alkane.

This approach enhances the reliability of compound identification by enabling comparison of experimentally determined RI values with literature data.

In cases where mass spectral matches did not agree with retention index values, compounds were conservatively classified as unidentified or tentatively assigned to broader chemical classes. Confidence levels of compound identification were interpreted in accordance with the framework proposed by Schymanski et al. (Level 2: probable structure identification) [41,42]. Although underivatized GC–MS analysis enabled comparative phytochemical profiling of the extracts, derivatization-based approaches such as BSTFA silylation may further improve the detection and characterization of polar metabolites in future studies [43]. Definitive structural elucidation of phytoconstituents would require complementary analytical approaches such as LC–MS/MS and NMR spectroscopy, which are recommended for future investigations.

Phytochemical profiling was carried out through GC–MS analysis, utilizing single injections for each sample. Solvent blanks were periodically used to check for background contamination, though a formal evaluation of carryover was not conducted. The analytical sequence did not include pooled quality control (QC) samples or replicate injections. Consequently, the findings are displayed as semi-quantitative profiles based on relative peak areas, without statistical variation.

### 3.8. GC–MS Compound Identification

Compound identification was carried out by comparing the obtained mass spectra with reference spectra from the NIST Mass Spectral Library. Identification was based on similarity index (match factor), retention index (RI) comparison, and consistency with reported literature data for non-polar columns (Rtx-5Sil/DB-5 type). Compounds with similarity index values ≥ 85% together with acceptable agreement between experimental and literature RI values (generally within ±10 RI units) were considered reliably identified, while those with lower similarity scores (79%) or lacking RI confirmation were reported as tentatively identified. Compounds with poor spectral matches or inconsistent RI values were classified as unidentified. Literature RI values were obtained from standard databases and references, including the NIST Chemistry Web Book and published compilations such as Adams (2017) [12], ensuring comparison with data obtained on comparable non-polar stationary phases.

## 4. Conclusions

This study provides comparative phytochemical and bioactivity information for leaf and fruit extracts of *Psidium cattleyanum*. Sequential solvent extraction combined with GC–MS analysis, quantitative phenolic and flavonoid determination, antioxidant assays, cytotoxicity evaluation, and qualitative phytochemical screening revealed clear differences in chemical composition and bioactive potential between the two plant parts. Leaf extracts exhibited significantly higher responses in total phenolic and flavonoid assays than fruit extracts, along with stronger antioxidant activity (IC_50_ = 47.10 ± 0.06 ppm) compared to fruit extracts (IC_50_ = 357.30 ± 0.64 ppm). Both extracts showed mild to moderate cytotoxicity (LC_50_ = 402.60–522.10 µg/mL), indicating the presence of biologically active phytochemicals warranting further investigation. GC–MS profiling further revealed that the leaf extract was comparatively enriched in sesquiterpenoid constituents, including caryophyllene, humulene, and cadinene-type compounds, whereas the fruit extract contained higher levels of lipid-derived constituents, particularly unsaturated fatty acid derivatives and phytosterol-related compounds. The enhanced antioxidant capacity of the leaf extract is consistent with its enrichment in phenolic compounds and terpenoid constituents, whereas the fruit extract displayed a comparatively distinct profile characterized by lipid-derived metabolites alongside tocopherol-related constituents. Overall, this study provides comparative phytochemical and bioactivity data that may support future targeted investigations of *P. cattleyanum,* highlighting the leaf as a comparatively rich source of antioxidant-associated phytochemicals under the conditions employed. These findings support further investigations involving bioassay-guided fractionation, compound isolation, and mechanistic studies to further evaluate the biological relevance and mechanisms of action of this species.

## Data Availability

The raw data supporting the conclusions of this article will be made available by the authors on request.

## Supplementary Materials

The following supporting information can be downloaded at: https://www.mdpi.com/article/10.3390/molecules31172984/s1, Table S1: Qualitative phytochemical screening of *Psidium cattleyanum* leaf and fruit extracts, Figure S1: Gallic acid standard calibration curve for TPC, Table S2: Absorbance values for the standard calibration curve of Gallic Acid for TPC, Figure S2: Total phenolic content (TPC) of *Psidium cattleyanum* leaf and fruit extracts expressed as mg GAE/g extract, Table S3: Absorbance values for 10 times diluted concentrations of *Psidium cattleyanum* leaf and fruit extracts for TPC, Figure S3: Catechin standard calibration curve for TFC, Table S4: Calculated total phenolic content with standard deviations for *Psidium cattleyanum* leaf and fruit extracts, Figure S4: Total flavonoid content (TFC) of leaf and fruit extracts expressed as mg CE/g extract, Table S5: Absorbance values for the standard calibration curve of Catechin for TFC, Figure S5: Standard ascorbic acid curve for the determination of IC50 value, Table S6: Absorbance values for 10 times diluted concentrations of *Psidium cattleyanum* leaf and fruit extracts, Figure S6: Potassium dichromate standard curve for the determination of the LC50 value, Table S7: Calculated total flavonoid content with standard deviations for *Psidium cattleyanum* leaf and fruit extracts, Table S8: Absorbance values for the standard Ascorbic Acid, Table S9: DPPH radical scavenging activity percentage inhibition for different concentrations of leaf and fruit extracts, Table S10: Mortality percentages for different concentrations of leaf and fruit extracts, Table S11: Mortality percentages for potassium dichromate standard curve, Table S12: Identified chemical compounds with their applications of *Psidium cattleyanum* leaf sample in GC–MS analysis, Table S13: Identified chemical compounds with their applications of *Psidium cattleyanum* fruit sample in GC–MS analysis. Table S14: GC–MS Instrumental Parameters. Table S15: n-Alkane series used for RI calibration (GC–MS). Figure S7: GC–MS total ion chromatogram (TIC) of the n-alkane standard series (C10–C33) analyzed under Shimadzu-recommended GC–MS conditions using an Rtx-5Sil MS column, used for retention index (RI) calibration.

## Abbreviations

The following abbreviations are used in this manuscript:

GC-MS	Gas chromatography–mass spectrometry
TPC	Total phenolic content
TFC	Total flavonoid content
DPPH	2,2-Diphenyl-1-picrylhydrazyl
GAE	Gallic acid equivalents
CE	Catechin equivalents
IC50	Half-maximal inhibitory concentration
LC50	Median lethal concentration
RI	Retention Index
BSTFA	N,O-bis(trimethylsilyl)trifluoroacetamide
LC-MS	Liquid chromatography–mass spectrometry
NMR	Nuclear magnetic resonance
NIST	National Institute of Standards and Technology
SD	Standard deviation
