# Supplementary material for: Integrated GC–MS Phytochemical Profiling and Bioactivity Assessment of Leaf and Fruit Extracts of Psidium cattleyanum (Cherry Guava)
Source: Molecules. 2026 Aug 26;31(17):2984. doi: 10.3390/molecules31172984 (PMC13566999; doi:10.3390/molecules31172984)
Supplement: Supplementary file 1 [file molecules-31-02984-s001.zip › molecules-4367356-supplementary.pdf]

Supplementary Materials

1. *Phytochemical screening*

Table S1. Qualitative phytochemical screening of *Psidium cattleyanum* leaf and fruit extracts

**Phytochemical Class**	**Test Performed**	**Leaf Extract**	**Fruit Extract**
Alkaloids	Meyer's test	+	–
Flavonoids	Shinoda test (Mg–HCl)	+	+
Phenols	Ferric chloride test	+	+
Tannins	Ferric chloride test	+	–
Saponins	Froth test	–	–
Terpenoids	Salkowski test	+	+
Steroids	Liebermann–Burchard test	–	+
Carbohydrates	Molisch test	–	–

Symbols: (+) Present; (–) Absent

2. *Total Phenolic Content (TPC)*

Table S2. Absorbance values for the standard calibration curve of Gallic Acid for TPC

**Gallic acid concentration (ppm)**	**Concentration in reaction mixture (ppm)**	**Absorbance at 765 nm**
Blank	Blank	0.000
10	0.5	0.049
20	1	0.095
40	2	0.192
80	4	0.312
160	8	0.695
320	16	1.129

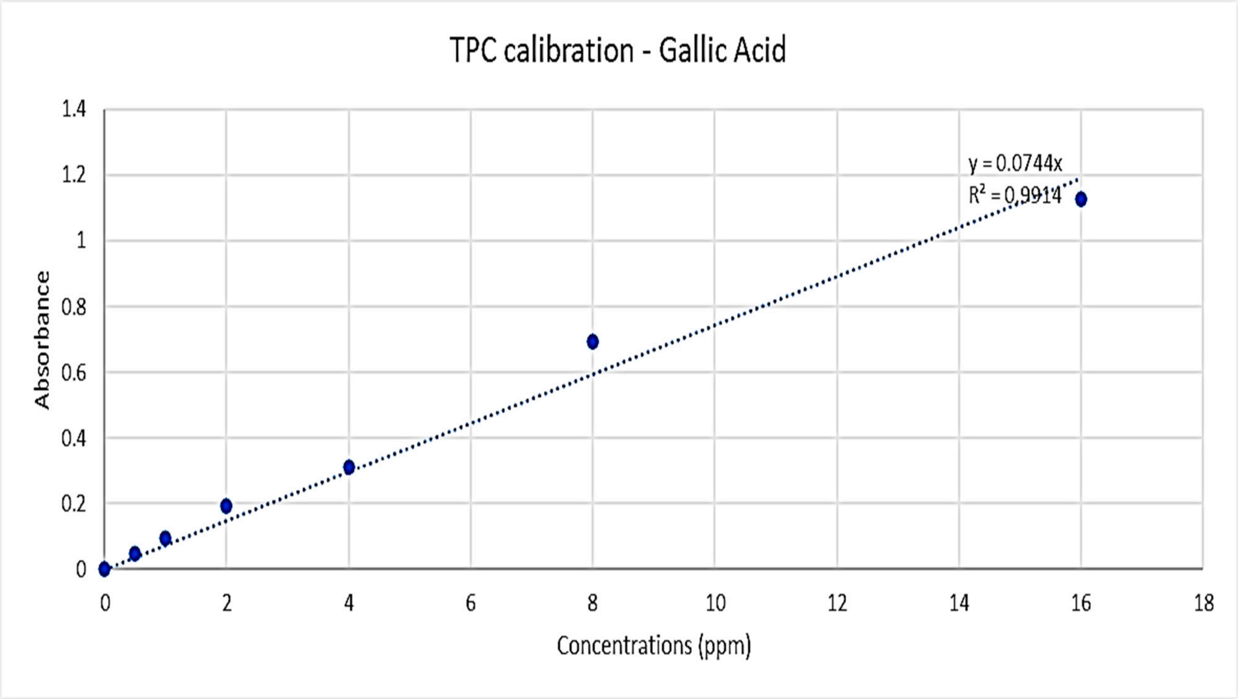

Figure S1. Gallic acid standard calibration curve for TPC. The intercept is forced to start from the origin.

Table S3. Absorbance values for 10,000ppm concentrations of *Psidium cattleyanum* leaf and fruit extracts for TPC

**Sample concentration (ppm)**	**Absorbance Values**	
	**Leaf Extract**	**Fruit Extract**
Dilute 1 - 10,000	1.191	0.333
Dilute 2 - 10,000	1.187	0.325

Table S4. Calculated total phenolic content with standard deviations for *Psidium cattleyanum* leaf and fruit extracts

**Sample**	**TPC (mg GAE/g dry extract)**	**SD**
Leaf	31.96	0.05
Fruit	8.87	0.11

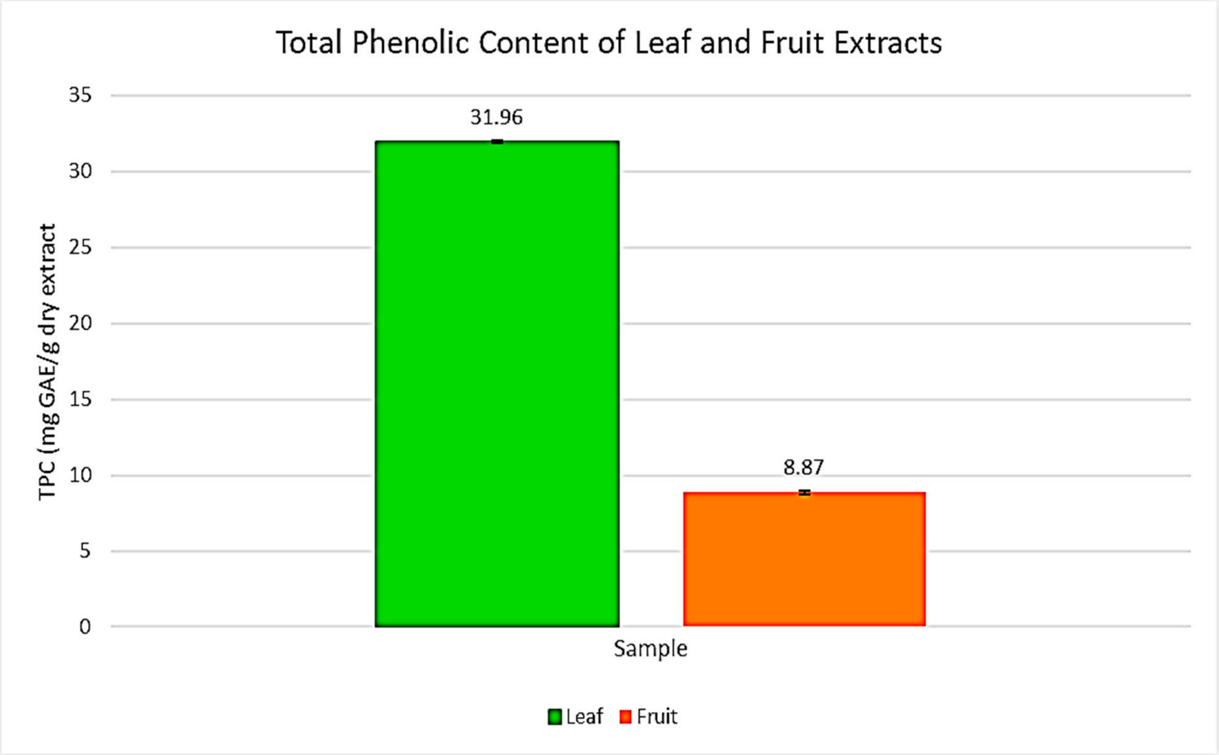

Figure S2. Total phenolic content (TPC) of *Psidium cattleyanum* leaf and fruit extracts expressed as mg GAE/g dry extract. Values represent mean ± SD (n = 2, analytical duplicates).

3. *Total Flavonoid Content (TFC)*

Table S5. Absorbance values for the standard calibration curve of Catechin for TFC

Standard series of Catechin (ppm)	Concentration in the reaction mixture (ppm)	Absorbance (A)
Blank	Blank	0.000
10	0.5	0.003
20	1	0.013
40	2	0.039
80	4	0.079
160	8	0.157
320	16	0.316

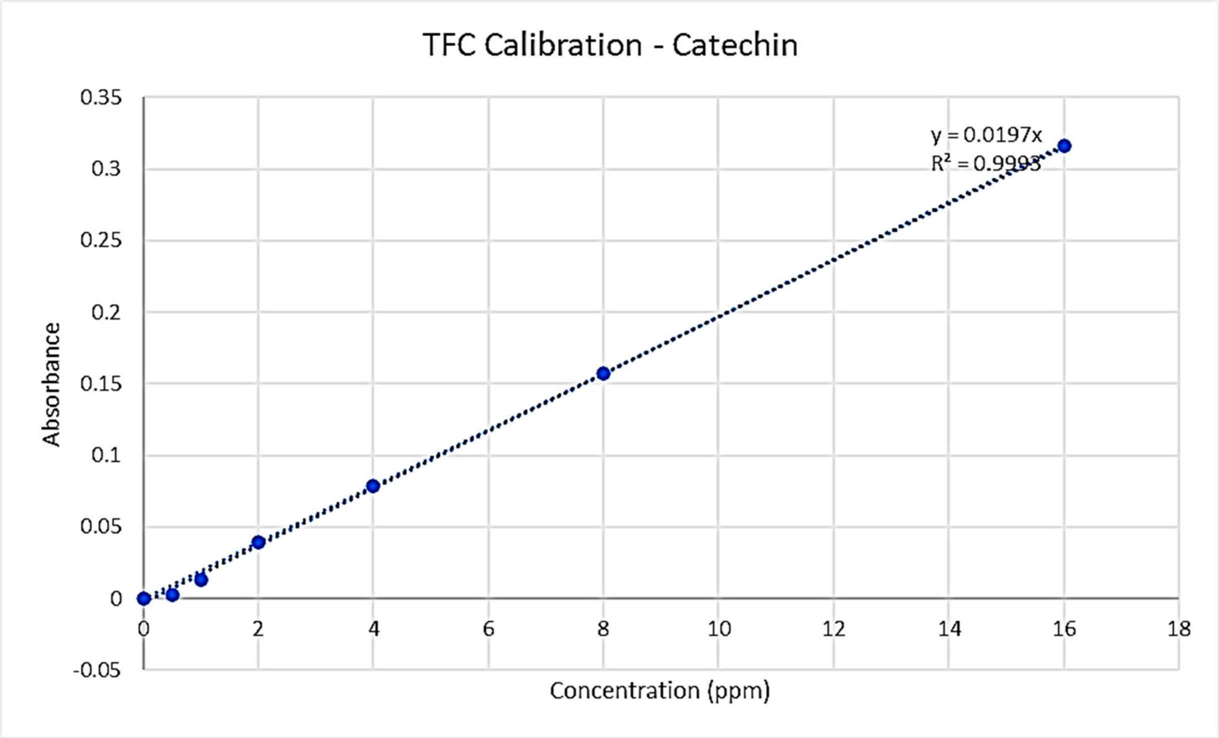

Figure S3. Catechin standard calibration curve for TFC. The intercept is forced to start from the origin.

Table S6. Absorbance values for 10,000ppm concentrations of *Psidium cattleyanum* leaf and fruit extracts

**Concentrations (ppm)**	**Absorbance**	
	**Leaf extract**	**Fruit extract**
Dilute 1 - 10,000	0.505	0.019
Dilute 2 - 10,000	0.508	0.02

Table S7. Calculated total flavonoid content with standard deviations for *Psidium cattleyanum* leaf and fruit extracts

**Sample**	**TFC (mg CE/g dry extract)**	**SD**
Leaf	51.43	0.22
Fruit	1.98	0.07[1]

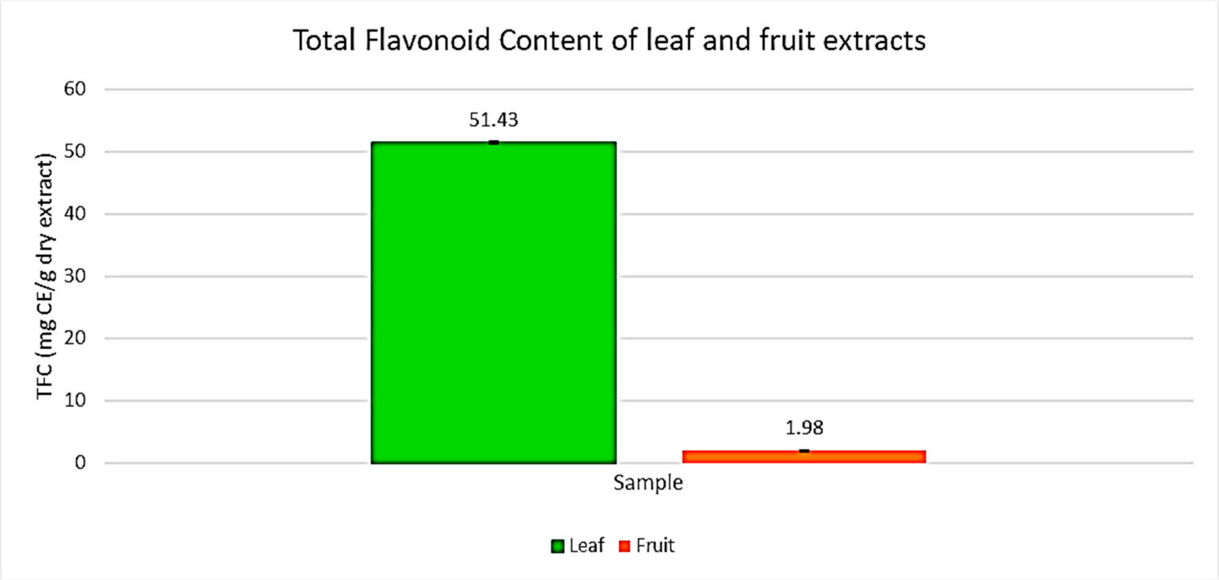

Figure S4. Total flavonoid content (TFC) of leaf and fruit extracts expressed as mg CE/g dry extract. Data represent mean ± SD (n = 2, analytical duplicates).

[1]The mass of dry extract used for TFC calculation was determined from the concentration of the dry extract reaction mixture and the volume added to the assay mixture.

The calibration curves for both the total flavonoid content (TFC) and total phenolic content (TPC) assays were reconstructed using the concentrations of the standards present in the final reaction mixtures after color development. The corrected calibration equations were then used to recalculate all reported TFC and TPC values

4. *Antioxidant Activity (DPPH Radical Scavenging Assay)*

Table S8. Absorbance values for the standard Ascorbic Acid

**Concentration (ppm)**	**Mean Absorbance**	**% Inhibition**
5	0.77 ± 0.08	2.12 ± 0.08
10	0.65 ± 0.06	13.62 ± 0.06
25	0.36 ± 0.04	51.61 ± 0.04
50	0.17 ± 0.03	77.27 ± 0.03
100	0.07 ± 0.07	90.54 ± 0.07
1000	0.04 ± 0.05	94.12 ± 0.05
200	0.06 ± 0.04	91.55 ± 0.04
500	0.05 ± 0.06	92.97 ± 0.06

Results are presented as means ± SD (n = 2, analytical duplicates)

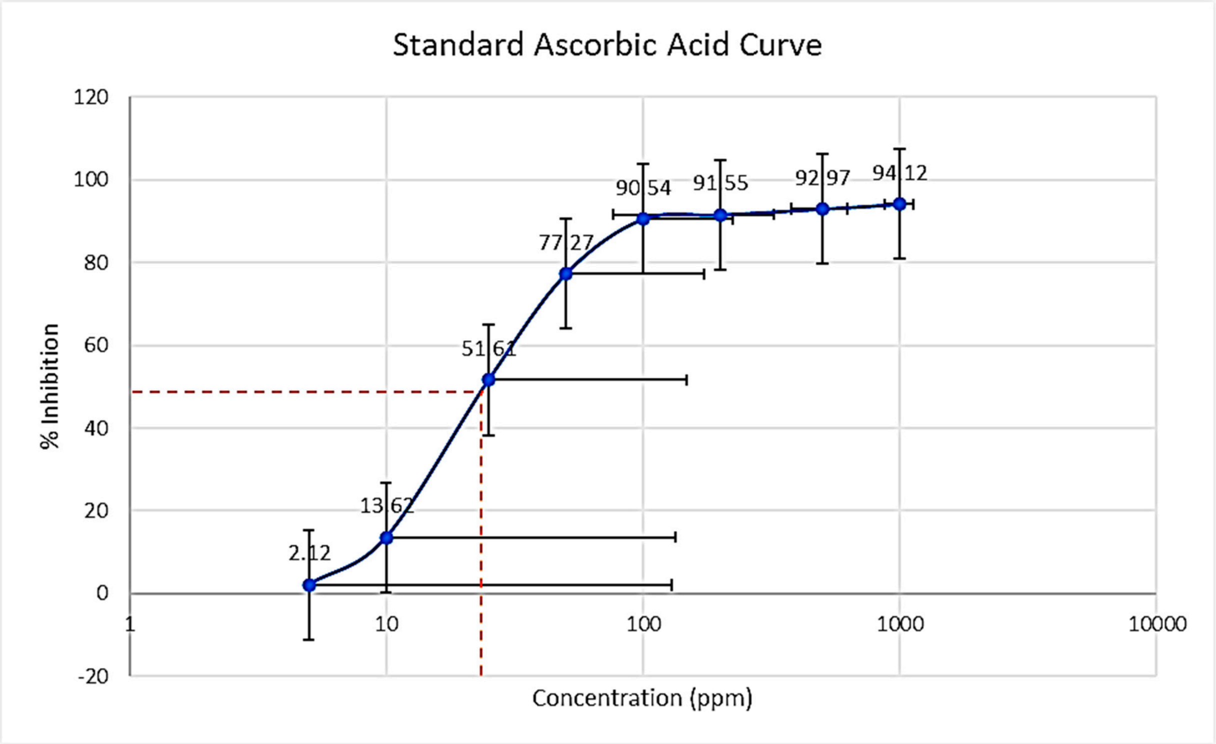

Figure S5. Standard ascorbic acid curve for the determination of IC_{50} value. Dotted lines interpret IC_{50} value of 23.9 ± 0.1 ppm for ascorbic acid.

Table S9. DPPH radical scavenging activity percentage inhibition for different concentrations of leaf and fruit extracts

Stock concentration (ppm)	Final Concentration (ppm)	Percentage Inhibition (%)	
		Leaf sample	Fruit sample
10,000	5,000	86.19 ± 0.05	85.22 ± 0.09
5,000	2,500	84.67 ± 0.05	83.84 ± 0.02
2,500	1,250	82.32 ± 0.09	82.60 ± 0.12
1,250	625	81.91 ± 0.08	82.46 ± 0.07
625	313	79.83 ± 0.03	80.94 ± 0.08
313	157	79.14 ± 0.08	42.27 ± 0.29
157	78	73.20 ± 0.03	4.97 ± 0.09
78	39	40.61 ± 0.04	3.32 ± 0.08

Results are presented as means ± SD (n = 2, analytical duplicates)

5. *Cytotoxicity Assessment by Brine Shrimp Lethality Bioassay*

Table S10. Mortality percentages for different concentrations of leaf and fruit extracts

**Concentration (µg/mL)**	**Fruit Extract % Mortality**	**Leaf Extract % Mortality**
1000	100.00 ± 0.00	100 ± 0.00
500	66.67 ± 1.67	46.67 ± 1.50
250	13.33 ± 0.94	20.00 ± 1.00
125	6.67 ± 0.47	0.00 ± 0.00
62.5	3.33 ± 0.47	0.00 ± 0.00
31.25	0.00 ± 0.00	0.00 ± 0.00

Results are presented as means ± SD (n = 3)

Table S11. Mortality percentages for potassium dichromate standard curve

**Concentration (µg/mL)**	**log C**	**Mortality (%)**	**Probit**
3.125	0.494850022	10	3.72
6.25	0.795880017	0	1.0334
12.5	1.096910013	40	4.75
25	1.397940009	90	6.28
50	1.698970004	100	8.9538
100	2	100	8.9538
200	2.301029996	100	8.9538

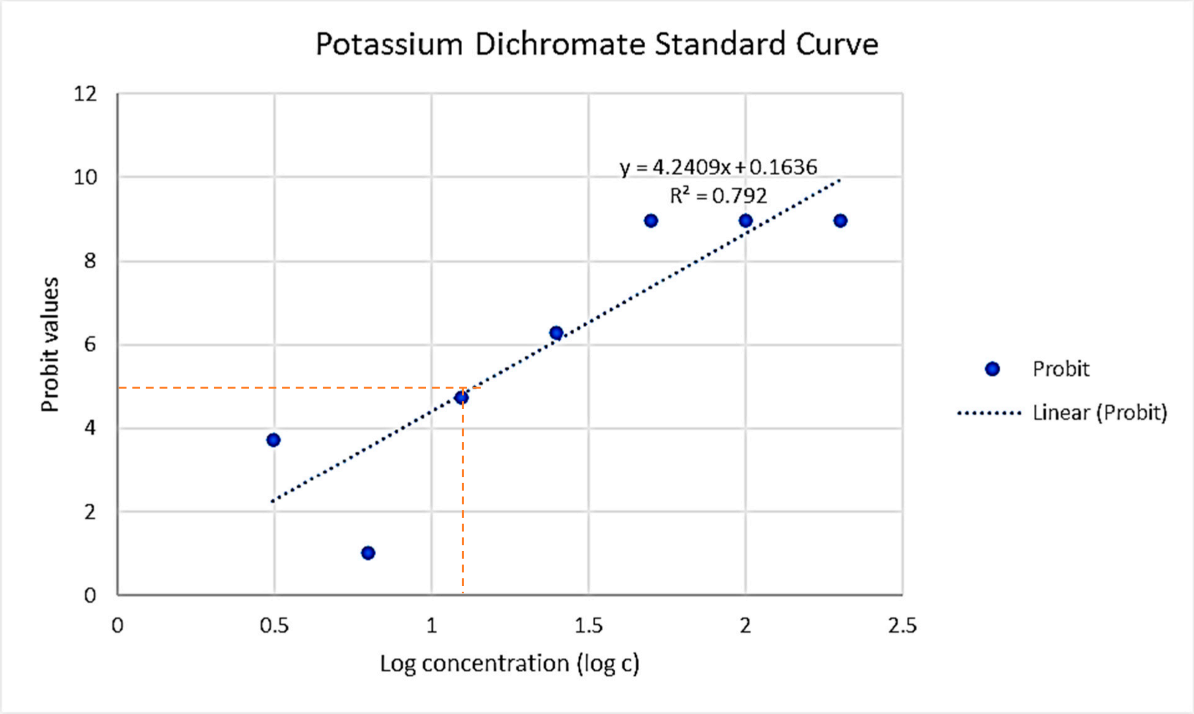

Figure S6: Potassium dichromate standard curve for the determination of the LC_{50} value. Probit analysis was performed to calculate LC_{50} values. Mortality (%) was converted to Probit units, plotted against log10(concentration), and linear regression was used to determine LC_{50} ± SD. Dotted line interpret the LC_{50} value in log C. (7.96 ± 0.68 µg/mL)

6. *GC–MS Profiling of Leaf and Fruit Extracts of P.cattleyanum.*

Supplementary Tables S12 and S13 present the detailed GC–MS compound lists for leaf and fruit extracts, including retention times and experimentally calculated retention indices (RI). Tentative identifications were based on NIST library matches and confirmed using calculated retention indices (RI).

Table S12. Identified chemical compounds with their applications of *Psidium cattleyanum* leaf sample in GC-MS analysis

Internal standard (IS): α-tocopheryl acetate (10 ppm); 500 μL added to 1 mL extract prior to GC–MS analysis.

Peak No.	RT (min)	Compound Identified	Similarity Search Index	Peak Area	Relative Abundance (%)	Semi-quantitative Concentration in the Injected Solution (μg/mL)*	RI (Cal)	RI (Lit)	ΔRI	Reported Biological Activities
1	7.308	Ylangene	94	325,775	0.17	0.017	1388	1373	+15	Antimicrobial, antioxidant, anti-inflammatory [15].
2	7.374	Copaene	87	1,415,920	0.75	0.074	1395	1390	+5	Anti-inflammatory [24]
3	7.877	Caryophyllene	97	25,285,969	13.42	1.319	1440	1435	+5	Sesquiterpene in essential oils; anti-inflammatory, antioxidant, analgesic [14].
4	8.242	Humulene	96	2,828,144	1.50	0.148	1473	1470	+3	Anti-tumor, anti-inflammatory, antimicrobial [14-17]
5	8.400	γ-Muurolene	93	1,104,096	0.59	0.058	1487	1478	+9	Antibacterial, antifungal, insecticidal, antioxidant [15].
6	8.650	α-Selinene	79	1,534,064	0.81	0.080	1491	1497	-6	Anti-inflammatory, antioxidant, antimicrobial [15].
7	8.850	β-Cadinene	86	2,693,409	1.43	0.141	1529	1524	+5	Antimicrobial, anti-inflammatory, antioxidant [15].
8	9.159	α-Maaliene	90	1,181,767	0.63	0.062	1558	1556	+2	Antimicrobial, antioxidant, anti-inflammatory; contributes to aroma and plant defense [15].
9	9.629	Caryophyllene oxide	92	1,699,081	0.90	0.089	1604	1583	+21	Bioactive sesquiterpene oxide; antifungal, anticancer [14-17].
10	10.155	Selina-6-en-4-ol	77	390,632	0.21	0.020	1652	1653	-1	Antioxidant and antimicrobial activity reported in essential oils [15].
11	10.205	τ-Cadinol	85	568,020	0.30	0.029	1657	1644	-7	Antifungal, antibacterial, cytotoxic

Peak No.	RT (min)	Compound Identified	Similarity Search Index	Peak Area	Relative Abundance (%)	Semi-quantitative Concentration in the Injected Solution (µg/mL)*	RI (Cal)	RI (Lit)	ΔRI	Reported Biological Activities
										against some cancer cell lines [15].
12	10.377	α-Cadinol	72	803,085	0.43	0.042	1674	1673	+1	Strong antimicrobial, antifungal, antioxidant, anti-inflammatory [15].
13	10.431	Neointermedeol	57	235,398	0.13	0.012	1679	1681	-2	Reported antimicrobial and anti-inflammatory activity [15].
14	13.016	Hexadecanoic acid, methyl ester	96	4,725,617	2.51	0.247	1930	1927	+3	Methyl palmitate; biodiesel, antioxidant, flavoring [25].
15	13.374	n-Hexadecanoic acid	90	1,963,749	1.04	0.103	1966	1968	−2	Antimicrobial, Emollient, surfactant, bioactive lipid [19]
16	14.697	6-Octadecenoic acid methyl ester, (Z)-	84	7,227,587	3.84	0.377	2108	2103	+5	Antimicrobial, lipid metabolite [19]
17	14.789	Phytol	91	3,930,746	2.08	0.205	2117	2118	−1	Diterpene alcohol; precursor for vitamins E & K; antimicrobial [20].
18	14.928	Methyl stearate	89	1,189,602	0.63	0.062	2132	2130	+2	Lipid metabolite [21]
19	15.050	9-Octadecenoic acid, (E)-	91	1,961,537	1.04	0.102	2145	2141	+4	Anti-inflammatory, hypolipidemic [21]
20	20.453	Squalene	97	7,475,639	3.96	0.390	2822	2818	+4	Precursor for cholesterol/steroids; antioxidant; cosmetic uses [21,22].
21	22.792	dl-α-Tocopherol	96	12,934,902	6.89	0.675	3151	3145	+6	Antioxidant; skincare and nutritional supplement [21,22].
22	23.182	α-Tocopheryl acetate (IS)	96	46,814,519	—	2.443	3191	3189	+2	Internal standard
23	24.645	γ-Sitosterol	90	5,617,479	2.98	0.293	3353	3350	+3	Phytosterol; cholesterol-lowering, anti-inflammatory [21,22].[2]

[2] Reported applications were assigned based on published literature and standard chemical references; for tentatively identified compounds, applications correspond to general uses of the respective chemical class. Relative abundance (%) was calculated based on the total integrated chromatographic peak area. The dl-α-tocopherol signal was carefully evaluated because a corresponding peak was detected in the α-tocopheryl acetate internal standard solution under the applied GC–MS conditions. Since dl-α-tocopherol was also detected in the original unspiked plant extracts, a possible internal standard-related artefactual contribution cannot be completely excluded. Therefore, the reported dl-α-tocopherol values should be interpreted as semi-quantitative estimates. Semi-quantitative concentrations in the injected analytical solution were estimated relative to the effective internal standard (α-tocopheryl acetate) using peak area normalization and are intended solely for comparative purposes.

Table S13. Identified chemical compounds with their applications of *Psidium cattleyanum* fruit sample in GC-MS analysis

Internal standard (IS): α-tocopheryl acetate (10 ppm); 500 μL added to 1 mL extract prior to GC–MS analysis.

Peak No.	RT (min)	Compound Identified	Similarity Search Index	Peak Area	Relative Abundance (%)	Semi-quantitative Concentration in the Injected Solution (μg/mL)*	RI (Cal)	RI (Lit)	ΔRI	Reported Biological Activities
1	7.094	Eugenol	97	128,278	0.17	0.011	1358	1362	+5	Antioxidant, antimicrobial [23]
2	7.374	α-Copaene	95	224,366	0.29	0.019	1395	1390	+5	Anti-inflammatory [24]
3	7.870	Caryophyllene	97	7,808,329	10.18	0.669	1439	1435	+4	Sesquiterpene in essential oils; anti-inflammatory, antioxidant, analgesic [14].
4	8.244	Humulene	93	1,139,108	1.49	0.098	1473	1470	+3	Anti-tumor, anti-inflammatory, antimicrobial [14-17]
5	8.399	γ-Muurolene	93	259,196	0.34	0.022	1487	1478	+9	Antimicrobial, antioxidant, anti-inflammatory, insecticidal; contributes to plant defense [15].
6	8.925	cis-Calamenene	81	620,090	0.80	0.053	1536	1529	+7	Strong antimicrobial (including antibacterial and antifungal), antioxidant, anti-inflammatory, insecticidal, and reported anticancer activity [15].
7	9.161	α-Maaliene	84	420,478	0.55	0.036	1558	1556	+2	Antimicrobial, antioxidant, anti-inflammatory; contributes to aroma and plant defense [15].
8	9.610	Caryophyllene oxide	94	124,550	0.16	0.011	1559	1583	-23	Bioactive sesquiterpene oxide; antifungal, anticancer [15-17].
9	10.064	Cubenol	87	381,290	0.49	0.033	1644	1645	+1	Antimicrobial, antioxidant, anti-inflammatory, insecticidal; contributes

Peak No.	RT (min)	Compound Identified	Similarity Search Index	Peak Area	Relative Abundance (%)	Semi-quantitative Concentration in the Injected Solution (μg/mL)*	RI (Cal)	RI (Lit)	ΔRI	Reported Biological Activities
1	7.094	Eugenol	97	128,278	0.17	0.011	1358	1362	+5	Antioxidant, antimicrobial [23]
										to essential oil bioactivity [15].
10	10.213	τ-Cadinol	75	560,095	0.73	0.048	1658	1644	+14	Potent antibacterial, antifungal, antioxidant, anti-inflammatory, and cytotoxic activity [15].
11	10.372	τ-Muurolol	86	435,160	0.57	0.037	1674	1664	+10	Antibacterial, antifungal, antioxidant, anti-inflammatory, insecticidal [15].
12	13.018	Hexadecanoic acid, methyl ester	96	3,330,274	4.34	0.285	1930	1927	+3	Methyl palmitate; biodiesel, antioxidant, flavoring [26].
13	13.378	n-Hexadecanoic acid	92	2,040,353	2.66	0.175	1967	1968	−1	Antimicrobial, Emollient, surfactant, bioactive lipid [20]
14	14.636	9,12-Octadecadienoic acid (Z,Z)-, methyl ester	96	2,547,604	3.32	0.218	2101	2098	+3	Anti-inflammatory, Nutraceutical, biodiesel, antioxidant [27].
15	14.696	8,11,14-Eicosatrienoic acid, methyl ester	85	5,530,286	7.21	0.474	2108	2105	+3	Anti-inflammatory [27]
16	14.790	Phytol	96	3,643,889	4.75	0.312	2117	2118	−1	Diterpene alcohol; precursor for vitamins E & K; antimicrobial [20].
17	14.930	Methyl stearate	87	755,642	0.98	0.065	2132	2130	+2	Lipid metabolite [19]
18	15.052	9-Octadecenoic acid, (E)-	90	1,741,710	2.27	0.149	2145	2141	+4	Anti-inflammatory, hypolipidemic [19]
19	15.252	Octadecanoic acid	88	575,561	0.75	0.049	2168	2165	+3	Emollient, lipid metabolite [19]
20	20.445	Squalene	95	390,949	0.51	0.034	2821	2818	+3	Precursor for cholesterol/steroids; antioxidant; cosmetic uses [21,22].
21	22.769	dl-α-Tocopherol	96	5,643,620	7.35	0.484	3148	3145	+3	Antioxidant; skincare and nutritional supplement [21,22].
22	23.158	α-Tocopheryl acetate (IS)	96	28,508,415	-	2.443	3188	3189	−1	Internal standard

Peak No.	RT (min)	Compound Identified	Similarity Search Index	Peak Area	Relative Abundance (%)	Semi-quantitative Concentration in the Injected Solution (µg/mL)*	RI (Cal)	RI (Lit)	ΔRI	Reported Biological Activities
1	7.094	Eugenol	97	128,278	0.17	0.011	1358	1362	+5	Antioxidant, antimicrobial [23]
23	24.610	γ-Sitosterol	88	2,820,659	3.67	0.242	3349	3350	−1	Phytosterol; cholesterol-lowering, anti-inflammatory [21,22].[3]

Table S14. GC–MS Instrumental Parameters

Parameter	Description
GC–MS system	Shimadzu 2010 GC–MS-QP2010 system (model as used)
Column	Rtx-5Sil MS (30 m × 0.25 mm i.d., 0.25 µm film thickness)
Carrier gas	Helium
Flow rate	1.69 mL/min
Injection volume	1 µL
Injection mode	Splitless
Injector temperature	250 °C
Oven temperature program	50.0°C (hold 1.00 min) → 300.0°C at 10.0°C/min (hold 38.50min)
Ionization mode	Electron impact (EI), 70 eV
Ion source temperature	200 °C
Quadrupole temperature	250 °C
Solvent cut time	5.00 min
Start time and end time	5.00 min – 45.00 min
Scan range	m/z 50–500

[3] Reported applications were assigned based on published literature and standard chemical references; for tentatively identified compounds, applications correspond to general uses of the respective chemical class. Relative abundance (%) was calculated based on the total integrated chromatographic peak area. The dl-α-tocopherol signal was carefully evaluated because a corresponding peak was detected in the α-tocopheryl acetate internal standard solution under the applied GC–MS conditions. Since dl-α-tocopherol was also detected in the original unspiked plant extracts, a possible internal standard-related artefactual contribution cannot be completely excluded. Therefore, the reported dl-α-tocopherol values should be interpreted as semi-quantitative estimates. Semi-quantitative concentrations in the injected analytical solution were estimated relative to the effective internal standard response (α-tocopheryl acetate) using peak area normalization and are intended solely for comparative purposes.

Table S15. n-Alkane series used for RI calibration (GC–MS)

**n-Alkane**	**Retention Time (min)**	**Retention Index (RI)**
C10	3.965	1000
C11	4.679	1100
C12	5.560	1200
C13	6.421	1300
C14	7.428	1400
C15	8.541	1500
C16	9.611	1600
C17	10.650	1700
C18	11.689	1800
C19	12.738	1900
C20	13.704	2000
C21	14.627	2100
C22	15.551	2200
C23	16.432	2300
C24	17.251	2400
C25	18.090	2500
C26	18.846	2600
C27	19.559	2700
C28	20.294	2800
C29	21.007	2900
C30	21.679	3000
C31	22.414	3100
C32	23.232	3200
C33	24.156	3300

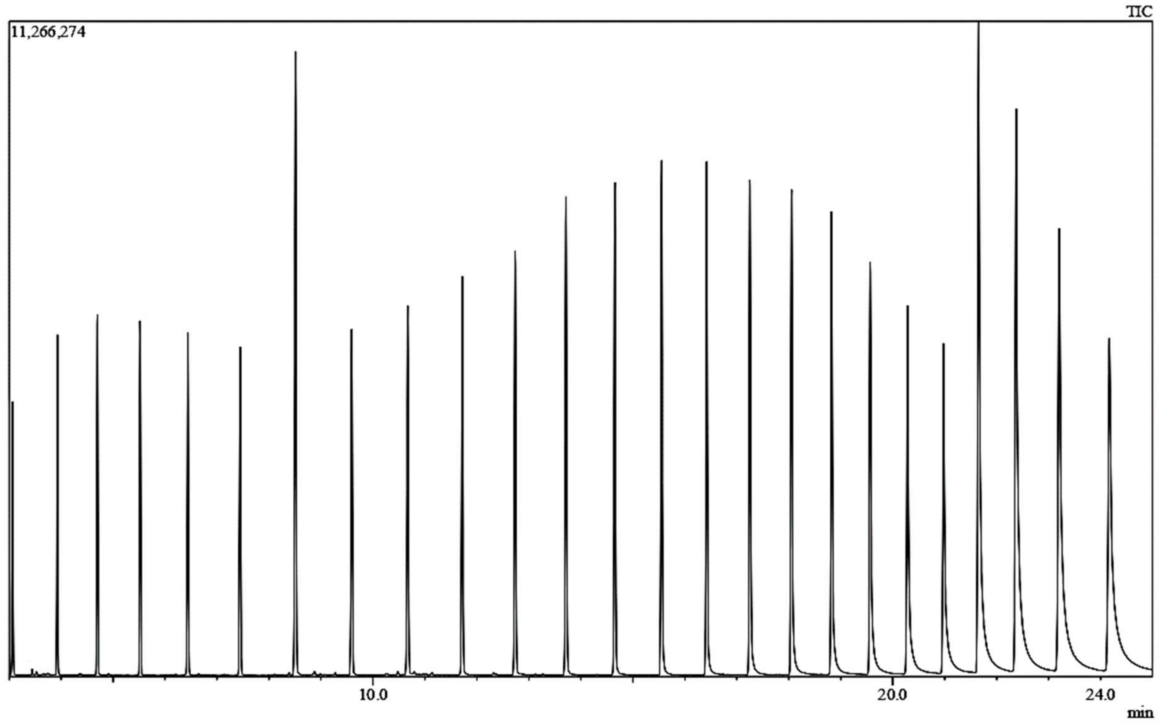

Figure S7: GC–MS total ion chromatogram (TIC) of the n-alkane standard series (C10–C33) analyzed under Shimadzu-recommended GC–MS conditions using an Rtx-5Sil MS column, used for retention index (RI) calibration.
